# Improvement of growth, yield and associated bacteriome of rice by the application of probiotic *Paraburkholderia* and *Delftia*

**DOI:** 10.3389/fmicb.2023.1212505

**Published:** 2023-07-14

**Authors:** Tofazzal Islam, Fatema, M. Nazmul Hoque, Dipali Rani Gupta, Nur Uddin Mahmud, Tahsin Islam Sakif, Andrew G. Sharpe

**Affiliations:** ^1^Institute of Biotechnology and Genetic Engineering (IBGE), Bangabandhu Sheikh Mujibur Rahman Agricultural University (BSMRAU), Gazipur, Bangladesh; ^2^Department of Gynecology, Obstetrics and Reproductive Health, BSMRAU, Gazipur, Bangladesh; ^3^Lane Department of Computer Science and Electrical Engineering, West Virginia University, Morgantown, WV, United States; ^4^Global Institute for Food Security, University of Saskatchewan, Saskatoon, SK, Canada

**Keywords:** probiotic bacteria, rice root, rhizosphere soil, microbiomes, microbial diversity

## Abstract

Plant probiotic bacteria enhance growth and yield of crop plants when applied at the appropriate time and dose. Two rice probiotic bacteria, *Paraburkholderia fungorum* strain BRRh-4 and *Delftia* sp. strain BTL-M2 promote growth and yield of plants. However, no information is available on application of these two bacteria on growth, yield, and diversity and population of bacteriome in roots and rhizosphere soils of the treated rice plants. This study aimed to assess the effect of BRRh-4 and BTL-M2 application on growth, yield and bacteriome in roots and rhizosphere soil of rice under varying doses of N, P and K fertilizers. Application of BRRh-4 and BTL-M2 strains significantly (*p* < 0.05) increased seed germination, growth and yield of rice compared to an untreated control. Interestingly, the grain yield of rice by these bacteria with 50% less of the recommended doses of N, P, and K fertilizers were statistically similar to or better than the rice plants treated with 100% doses of these fertilizers. Targeted amplicon (16S rRNA) sequence-based analysis revealed significant differences (PERMANOVA, *p* = 0.00035) in alpha-diversity between the root (R) and rhizosphere soil (S) samples, showing higher diversity in the microbial ecosystem of root samples. Additionally, the bacteriome diversity in the root of rice plants that received both probiotic bacteria and chemical fertilizers were significantly higher (PERMANOVA, *p* = 0.0312) compared to the rice plants treated with fertilizers only. Out of 185 bacterial genera detected, *Prevotella*, an anaerobic and Gram-negative bacterium, was found to be the predominant genus in both rhizosphere soil and root metagenomes. However, the relative abundance of *Prevotella* remained two-fold higher in the rhizosphere soil metagenome (52.02%) than in the root metagenome (25.04%). The other predominant bacterial genera detected in the rice root metagenome were *Bacillus* (11.07%), *Planctomyces* (4.06%), *Faecalibacterium* (3.91%), *Deinococcus* (2.97%), *Bacteroides* (2.61%), and *Chryseobacterium* (2.30%). On the other hand, rhizosphere soil metagenome had *Bacteroides* (12.38%), *Faecalibacterium* (9.50%), *Vibrio* (5.94%), *Roseomonas* (3.40%), and *Delftia* (3.02%). Interestingly, we found the presence and/or abundance of specific genera of bacteria in rice associated with the application of a specific probiotic bacterium. Taken together, our results indicate that improvement of growth and yield of rice by *P. fungorum* strain BRRh-4 and *Delftia* sp. strain BTL-M2 is likely linked with modulation of diversity, structures, and signature of bacteriome in roots and rhizosphere soils. This study for the first time demonstrated that application of plant growth promoting bacteria significantly improve growth, yield and increase the diversity of bacterial community in rice.

## Introduction

The current agricultural system is heavily dependent on the intensive use of chemical fertilizer and pesticides. However, application of synthetic chemical inputs in agriculture has negative effects on water, soil, and human and animal health (Nicolopoulou-Stamati et al., 2016; Hirt, 2020). Rice is the staple food of Bangladesh and many Asian and African countries and high-yielding rice varieties require extensive application of synthetic chemical fertilizers such as nitrogen (N), phosphorus (P) potassium (K) for achieving higher yield for ensuring food security on these continents (Hazell, 2010). Bangladesh consumes approximately 6 million metric tons of chemical fertilizers annually for rice and other crop cultivation, of which about 80 percent are imported (Rahman et al., 2018b). The four major imported chemical fertilizers in Bangladesh are urea, triple super phosphate, diammonium phosphate, and muriate of potash (MOP; Rahman et al., 2018b; Singh et al., 2021). To maintain the substantial increase in rice yield to feed the ever increasing population of rice growing countries, farmers need to apply higher doses of these fertilizers to the field (Rahman et al., 2018b). A large proportion of the applied synthetic chemical fertilizers are lost in various ways and pollute the environment (Rahman et al., 2018a; Singh et al., 2021). Sadly, due to the deterioration of soil health by the use of synthetic fertilizers over a long time, the yields of rice crops have not increased as they have increased before (Singh, 2018). Furthermore, to offer fertilizers at an affordable price to farmers, the government of Bangladesh spends huge funds every year subsidizing them. The natural mineral sources of these chemical fertilizers are also finite. Therefore, demand remains for searching alternative ways that would help to promote growth and increase yield of rice. Plant probiotic bacteria have shown high promise in significantly reducing the dependency on synthetic chemical fertilizers for growth and yield of rice (Trân Van et al., 2000; Khan et al., 2016; dos Santos et al., 2020). Application of these renewable bioresources could also reduce production costs and improve soil health in a sustainable manner.

The microbial world is the largest unexplored reservoir of biodiversity, which may act as a major benefit for agriculture, industrial and medicinal applications (Maheshwari, 2011; Geisen et al., 2019). One of the potential ecologically and economically sustainable strategies for the reduction of the dependence on chemical fertilizers could be the discovery of plant probiotic bacteria from native plants and use them as biofertilizer and/or biostimulants (Islam and Hossain, 2012; Islam et al., 2016). Application of an appropriate plant probiotic bacterium or a consortium of elite probiotic bacteria has been found to significantly enhance growth and yield of crop plants with significant reduction of the requirements for chemical fertilizers (Islam et al., 2016; Menéndez and Paço, 2020). A large number of plant probiotic bacterial genera exist, such as *Bacillus*, *Pseudomonas*, *Paraburkholderia*, *Klebsiella*, *Rhizobium*, *Rahnella*, *Enterobacter*, *Delftia*, *Azospirillum*, *Streptomyces,* and they have been used in commercial formulation as biofertilizer in many countries, which are now a multibillion-dollar business worldwide (Zambrano-Mendoza et al., 2021). In addition to growth promotion through diverse direct and indirect mechanisms, they also protect plants from pathogens by producing antimicrobial substances and enhance stress resistance to plants by inducing the expression of stress-responsive genes in the host plant (Harman et al., 2001; Zambrano-Mendoza et al., 2021). Plant probiotic bacteria that express aminocyclopropane-1-carboxylic acid (ACC) deaminase activity protects plants from growth inhibition by flooding and anoxia, drought, high salt, the presence of fungal and bacterial pathogens, nematodes, and the presence of metals and organic contaminants (Gamalero and Glick, 2011; Kandel and Kandel, 2015). To promote plant-growth, probiotic bacterial inoculants must either establish themselves in the soil or become associated with the host plant. The effects of microbial inoculation could be positive or negative depending on the microflora of root and rhizosphere (Ambrosini et al., 2016). Since only about 5% of these microorganisms are cultivable, the effects of the application of probiotic bacteria on the microbial diversity and population remained vastly unknown. Better understanding of the effect of applied plant growth promoting probiotic bacteria (PGPB) on the change in diversity and populations of the native bacteria in roots and rhizosphere are important for better understanding of the tripartite interactions which is critical for their sustainable application in crop production.

Metagenomics analysis opens a new window to explore the microbial diversity of various ecosystems without direct culturing of the microorganisms (Hoque et al., 2021; Rafiqul Islam et al., 2022). This approach is becoming increasingly popular in large-scale genomics applications as a way to study the taxonomic and functional composition of microbial communities from environmental, agricultural, and clinical settings (French et al., 2021; Rahman et al., 2021; Hoque et al., 2022). Metagenomics can improve our understanding of the importance of microbes to plants and the associations that exist among them (Melcher et al., 2014) that can be used for better understanding the complex interactions between crop plants and soil microbiome critical for crop production (Melcher et al., 2014; Trivedi et al., 2017). Novel antibiotics and enzymes are among the early discoveries from metagenomics. This state-of-the-art technology is also now being applied to identify unknown pathogen(s) in outbreaks of emerging and re-emerging diseases (Hoque et al., 2019, 2021; Rafiqul Islam et al., 2022). Targeted 16S/18S/ITS amplicon sequencing is a powerful and affordable tool for clinical microbiota analysis (Islam et al., 2021). It can also be used to determine gut microbial species and their abundance, and allows to the monitoring of human health and well-being. Moreover, metagenomics can support the development of probiotics (Xu et al., 2019).

Our previous studies showed that the application of PGPB such as *Paraburkholderia* spp. strain BRRh-4 and *Delftia* sp. strain BTL-M2 significantly improves plant growth and increases grain yield of rice under reduced application of N, P, and K fertilizers (Khan et al., 2016). So far, the known mechanisms of these two bacteria in plant growth promotion are the production of indole-3-acetic acid (IAA), fixation of atmospheric nitrogen and solubilization of essential mineral nutrients such as phosphorus and potassium (Khan et al., 2016). However, detailed information is not available on whether the application of plant probiotic *Paraburkholderia* sp. and *Delftia* sp. modulate diversity and population of bacteria in roots and rhizosphere soils of the treated rice plants. Therefore, we hypothesized that the application of probiotic bacterial strains, BRRh-4 and BTL-M2 improves growth and yield of rice under nutrient deficient soils by improving the diversity of the bacteriome in roots and rhizosphere soils of rice. To test this hypothesis, we evaluated the effects of *P. fungorum* strain BRRh-4, and *Delftia* sp. strain BTL-M2 on growth, yield of rice under varying fertilizer doses and analysed the diversity and population of the bacterial community in rice roots and rhizosphere soils using the targeted 16S rRNA gene metagenomics sequencing.

## Materials and methods

### Rice seeds and probiotic bacterial strains

Seeds of BRRI dhan49 were collected from Bangladesh Rice Research Institute (BRRI), Joydebpur, Gazipur. This variety is cultivated in the Aman season (July to December) in Bangladesh. Two previously characterized rice growth promoting probiotic bacterial strains *viz. P. fungorum* strain BRRh-4 (Rahman et al., 2018a) and *Delftia* sp. strain BTL-M2 (Rahman et al., 2018a) were used in this study.

### Seedling assay

Bacterial strains, BRRh-4 and BTL-M2 were cultured in 250 mL conical flasks containing 200 mL NB (Nutrient Broth) medium on an orbital shaker at 120 rpm for 72 h at 27° C. The bacterial cells from broth were collected by centrifugation at 1,500 rpm for 1 min at 4°C and washed twice with SDW (Sterilized Distilled Water). The bacterial pellets were suspended in 0.6 mL SDW and mixed thoroughly by vortexing for 45 s before using for the seed treatment. Ten gram of surface-sterilized rice seeds cv. BRRI Dhan49 was soaked into bacterial suspension (1 × 10^9^ CFU/mL). The bacteria treated seeds were then dried overnight at room temperature to ensure better coating of the seeds with the bacteria. The treated seeds were placed on a Petri dish containing a water-soaked sterilized filter paper. After seed germination, the seedlings were allowed to grow for 10 days and 15 days. The seedlings were watered on every alternate day (Mahanta et al., 2022). The germination percentage was calculated in the seedling assay test both in the probiotic treatments and untreated control seeds at two DAI (days after inoculation). To assess the effects of plant probiotic bacteria on the growth of rice seedlings, data were collected twice at 10 DAI and 15 DAI. The following parameters were recorded - shoot length (cm), root length (cm), shoot fresh weight (mg), shoot dry weight (mg), root fresh weight (mg) and root dry weight (mg).

### Effect of probiotic bacteria on rice growth and yield

A pot experiment was conducted in a completely randomized design with three replications for each treatment. The treatments of the experiment were as follows: (i) zero dose of chemical fertilizer control; (ii) 50% dose of recommended chemical fertilizers (N as urea, P as TSP and K as MoP); and (iii) 100% dose of recommended chemical fertilizers with or without the treatment of either BRRh-4 or BTL-M2 (Karim et al., 2021). In this study, zero dose means no chemical fertilizers used; half dose indicates 50% of recommended N, P, and K fertilizers used and full dose means 100% of recommended N, P, and K fertilizers used. The soil of the experimental pots belonged to the Salna series in the Madhupur tract (AEZ 28) under the order Inceptisol (Khan et al., 2017), is a dark grey colour with clay loam having a pH of 5.5 (Kumar et al., 2019). Final soil preparation for the experiment was done while well rotten cow dung was applied in adequate quantity before layout preparation to supplement organic matter to the soil. Chemical fertilizers were applied as following recommended doses. For 100% doses, urea 61.75 g, triple super phosphate (TSP) 68.02 g, muriate of potash (MoP) 42.05 g, gypsum 31.63 g, and zinc (mono) 2.75 g, were used in each pot. In 50% doses, urea 30.875 g, TSP 34.01 g, MoP 21.02 g, gypsum 15.81 g, and zinc 1.375 g were used. All TSP, MoP, gypsum and zinc were used at the time of soil preparation as basal dose. Urea was applied at three time points; first point at 12 days after transplanting, second point at the time of tillering (25–30 days after transplanting), and a third point at the time of booting (40–45 Days after transplanting; Khan et al., 2016; Singh et al., 2021). The seeds were placed on a pot containing sterilized soils as the control replications. After seed germination, the seedlings were allowed to grow for 30 days. Seeds of BRRI dhan49 were sown directly in the nursery bed at the BSMRAU Farm. The seedbed was lightly irrigated regularly for ensuring proper growth and development of the seedlings. Thirty-day old seedlings (3/4-leaf stage) were transplanted in the well-prepared experimental pots. A total of 54 seedlings were transplanted in pots at the rate of 2 seedlings per pot.

Bacterial strains, BTL-M2 and BRRh-4 were cultured in 1,000 mL NB in a conical flask taking a single colony from the regularly maintained bacterial culture plate. Then the flask was placed on a shaking incubator adjusted at 120 rpm and 25^o^ C for 72 h for bacterial growth in NB. The bacterial suspension was then serially diluted to a concentration of 1 × 10^9^ CFU/ mL with sterile distilled water. Before transplanting, seedlings were uprooted, washed and the seedling roots were dipped overnight in such bacterial suspension (1 × 10^9^ CFU/mL) for better bacterial root colonization. Cultural operations like irrigation and weeding were done when required. Plant growth parameters such as plant height (cm), number of tillers and effective tillers per plant, filled and unfilled grains, 1,000 grain weight (g) and grain yield (g) per pot were recorded during harvesting time following method described by Khan et al. (2016).

### Metagenomics analysis of rice root and rhizosphere soil

#### Sample collection

The rhizospheric soil and rice root samples were separately collected from the bacteria treated and untreated rice plants. The washed and surface-sterilized root samples were free from the soil.

### Extraction of genomic DNA, metagenomic library preparation and sequencing

Genomic DNA was separately extracted from the collected rhizospheric soil and pre-pulverized rice roots using Maxwell plant DNA extraction kit (Promega Corporation, Madison, United States) and PowerSoil DNA Total Isolation Kit (MOBIO Laboratories Inc. Solana Beach CA, United States) following the manufacturer’s protocol. Extracted DNA was quantified with a NanoDrop2000 spectrophotometer (Thermo Scientific, United States). The DNA was stored at −20°C for further use. For gene library construction, the bacterial 16S rRNA gene was amplified by PCR with a set of primers (338F; 5´-ACTCCTACGGGAGGCAGCAG-3′ and 806R; 5´-GGACTACHVGGGTWTCTAAT-3′) targeting the hypervariable V3-V4 regions (about 460 bp; Dong et al., 2019; Hoque et al., 2020). The PCR reactions were as follows: initial denaturation: 3 min at 94°C, 30 cycles of 30 s at 94°C (denaturation), 30 s at 62°C (annealing) and 45 s at 70°C (extension) followed by 7 min at 72°C as final extension. PCR products were purified by Agencourt AMPure XP (Beckman Coulter). Samples were measured by NanoDrop2000, Qubit and Agilent 2,100 Bioanalyzer (Agilent Technologies). PCR amplification to introduce Illumina index sequences was performed in PCR strip tubes in a BioRad T100 thermocycler. The library DNA fragments were size selected and purified using AMPure XP beads (Beckman Coulter, Inc.). The indexed libraries were normalized, pooled and loaded onto an Illumina iSeq100 reagent cartridge using iSeq100 reagent kit v3 and 600 cycles. The paired-end 2 × 300 bp sequencing was run on an Illumina iSeq100 platform (Illumina, CA, United States) at Shanghai Majorbio Bio-Pharm Technology Co., Ltd.

### Data processing, OTU clustering and microbial community analysis

The Illumina generated reads were initially quality checked with FASTQC v.0.11.552 (Brown et al., 2017). Then the resulting FASTQ files were concatenated, filtered and end-trimmed with BBDuk (with options k = 21, mink = 6, ktrim = r, ftm = 5, qtrim = rl, trimq = 20, minlen = 30, overwrite = true) to remove Illumina adapters, known Illumina artifacts, and phiX (Hoque et al., 2019). Any sequences below these thresholds or reads containing more than one ‘N’ were discarded. The clean high-quality single-end reads were then denoised, and clustered to collapse into similar sequences (e.g., those that were ≥ 97% similar to each other) into single replicate sequences in the Quantitative Insights into Microbial Ecology (QIIME) version 2 (Bolyen et al., 2019) using DADA2 package (Callahan et al., 2016). Sequences were clustered into Operational Taxonomic Units (OTUs) with QIIME2 search cluster-features-closed-reference OTU algorithm with 99% similarity to the Greengenes Database version 13.8 (McDonald et al., 2012). Representative sequences from each OTU were assigned down to the phylum, order and genus level using the trained Naive Bayes classifier and the q2-feature-classifier plugin to the Greengenes Database (McDonald et al., 2012). Finally, the OTU table was saved, and OTUs accounting for less than 0.005% of the total sequences were removed (Bokulich et al., 2018).

### Statistical analysis

The SPSS version 16 was used for analysis of variance (ANOVA) of the data collected from the seedling assay and pot experiments. Treatment means were separated using Fisher’s protected LSD test at (*p* ≤ 0.05) For the metagenomics analysis, the Shapiro–Wilk test was used to check the normality of the data, and the non-parametric test Kruskal-Wallis rank-sum test was used to evaluate differences in the relative abundance of bacterial taxa at different levels according to metagenome groups. Alpha diversity (diversity within samples) was estimated using the observed species and Shannon diversity indices for QIIME read assignments and counts in root and rhizosphere soil, chemical fertilizer (*CF*), *CF* + *Delftia*, and *CF* + *Paraburkholderia* treated samples. Beta diversity was calculated for the microbiota of root and rhizosphere soil, chemical fertilizer (*CF*), *CF* + *Delftia*, and *CF* + *Paraburkholderia* treated metagenomes using Bray–Curtis dissimilarity (Clarke et al., 2006), and PERMANOVA (permutational multivariate analysis of variance using distance matrices; Anderson and Walsh, 2013). Principal component analysis (PCA) was used to visualize differences between microbial communities. Only OTUs with an abundant presence (>0.1% of total reads in at least one sample) were included in the analysis (Fernández-González et al., 2019).

## Results

### Probiotic bacteria, *Paraburkholderia*, and *Delftia* promote rice seed germination

The seed germination rate was calculated 3 days after bacterial inoculation. The germination percentage was significantly higher (*p* < 0.050, One-way ANOVA) in the probiotic treated seeds compared to the untreated control. The highest germination percentage (89.23%) was recorded in BTL-M2 (*Delftia*), followed by BRRh-4 (*P. fungorum*; 87.69%) and untreated controls (76.92%; Table 1).

**Table 1:** Germination rate of the seeds after probiotic treatment.

Treatments	Total number of seeds	Number of seeds germinated	Number of seeds not germinated	Germination percentage (%)
BTL-M2	65	58	7	89.23
BRRh-4	65	57	8	87.69
Control	65	50	15	76.92

### Probiotic bacteria improve shoot and root length of rice seedlings

**Figure 1:**
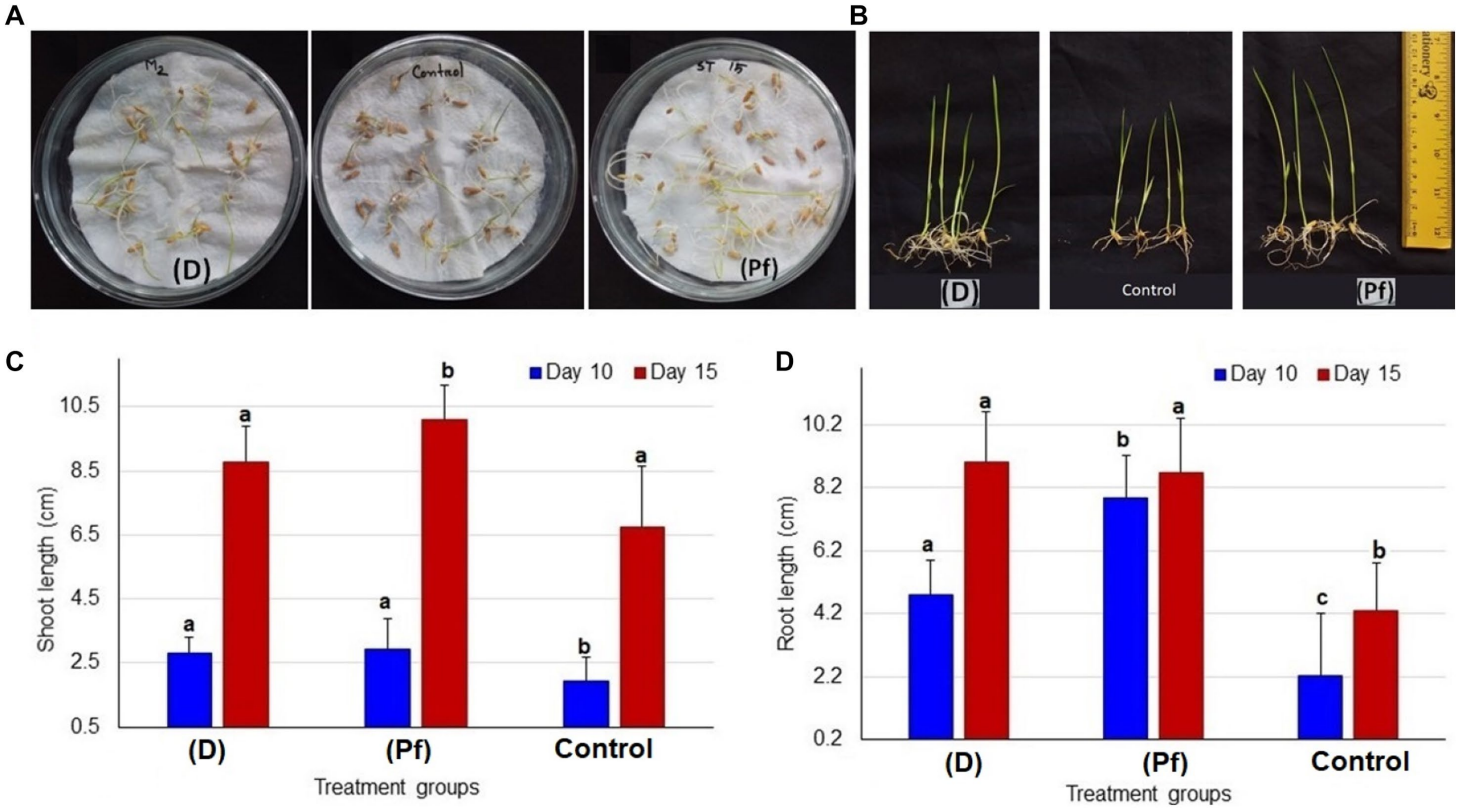
Comparative growth of seedlings between two different probiotic treatments and untreated controls. **(A)** Comparative growth at 5 days after inoculation (DAI); seedlings treated with BTL-M2 (D; *Delftia*), untreated control seedlings and seedlings treated with BRRh-4 (Pf; *P. fungorum*). **(B)** Proportional view of seedlings among the probiotic treatments (*Delftia* and *P. fungorum*) and untreated control plants at DAI 15. **(C)** Effects of Delftia and P. fungorum inoculation on the shoot length of the rice seedlings at DAI 10 and DAI 15. **(D)** Effects of *Delftia* and *P. fungorum* inoculation on the root length of the rice seedlings at DAI 10 and DAI 15.

The root and shoot growth of rice seedlings significantly (*p* < 0.05, One-way ANOVA) varied by the effects of probiotic bacteria at 5 days after inoculation (DAI; Figure 1A), and 15 DAI (Figure 1B). The highest average shoot length was observed in the rice seedling obtained from the seeds treated with BRRh-4 at 10 DAI (3.0 ± 0.95 cm, mean ± SD) and at 15 DAI (10.1 ± 1.08 cm; Figure 1C). Conversely, the lowest shoot length was recorded in the untreated control plant both at 10 DAI (1.95 ± 0.44 cm) and 15 DAI (6.76 ± 1.19 cm; Figure 1C). Similarly, the root lengths of the studied rice seedlings also varied significantly when obtained from seeds treated with the probiotic bacterial isolates (Supplementary Figure S1). The highest average root length was recorded in the plant treated with BRRh-4 at 10 DAI (7.85 ± 1.35 cm), however, at 15 DAI the highest root length was found in the plant treated with BTL-M2 (9.0 ± 1.62 cm; Figure 1D).

### Effects of probiotic bacteria on shoot weight of rice seedlings

**Figure 2:**
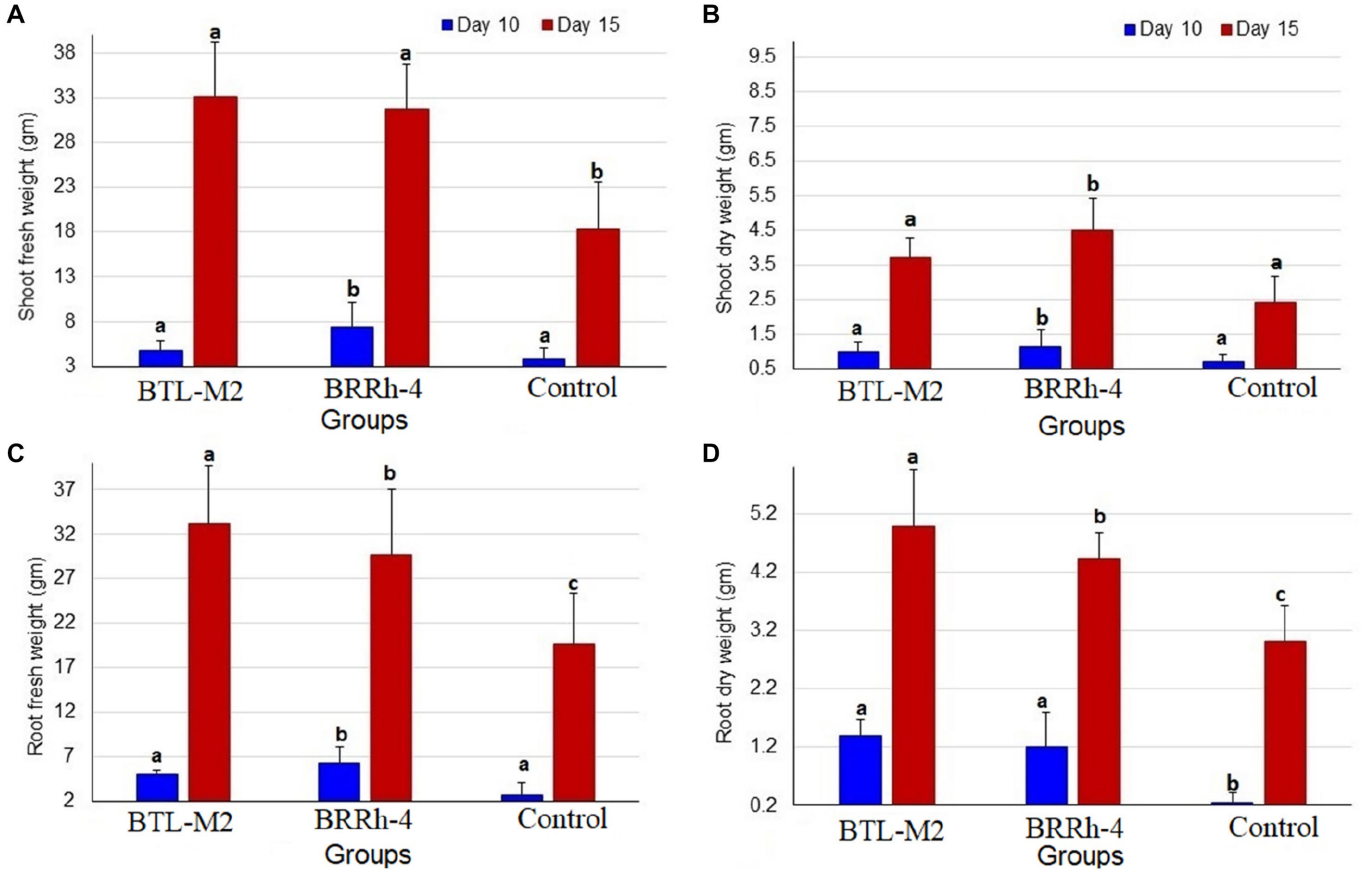
Graphical representation of shoot and root weight of rice seedlings. **(A)** Comparative shoot fresh weight of BTL-M2(*Delftia*), BRRh-4 (*P. fungorum*) and untreated control plants at 10 DAI and 15 DAI. **(B)** Comparative shoot dry weight of BTL-M2and BRRh-4 treated plants and untreated control plants at 10 DAI and 15 DAI. **(C)** Comparative root fresh weight of BTL-M2and BRRh-4 treated plants and untreated control plants at 10 DAI and 15 DAI. **(D)** Comparative root dry weight of BTL-M2and BRRh-4 treated plants and untreated control plants at 10 DAI and 15 DAI. Error bars represent the standard deviation (SD). Superscripts (a, b, and c) show significant differences in different parameters on the shoot and root growth among the treatment groups (one-way ANOVA test, *p* < 0.05).

The bacterial strains, BTL-M2 and BRRh-4 inoculation increased the shoot weight of rice seedlings positively in the case of shoot fresh weight at 10 DAI. In this study, the highest average mean weight was recorded in the plants treated with BRRh-4 (7.41 ± 2.8 mg) compared to that of BTL-M2 (4.82 ± 0.7 mg) and the untreated control (3.83 ± 1.19 mg) plants (Figure 2A). However, at 15 DAI, the highest average shoot fresh weight was found in the plants treated with BTL-M2 (33.07 ± 6.18 mg) compared to BRRh-4 treated (31.82 ± 4.9 mg) and untreated control plants (18.27 ± 0.6 mg; Figure 2A). Similarly, in the case of shoot dry weight, the highest average weight was recorded in plants treated with BRRh-4 both at DAI 10 (1.16 ± 0.48 mg) and DAI 15 (4.5 ± 0.92 mg), which was statistically dissimilar with the untreated control plants. Seedling assay revealed that the lowest average weight was recorded in the untreated control plants both at DAI 10 (0.7 ± 0.24 mg) and DAI 15 (2.33 ± 0.74 mg; Figure 2B). The selected bacterial inoculation also showed a positive effect on the root weight of rice seedlings (Figure 2).

At DAI 10, the highest average root fresh weight was recorded in the plants treated with BRRh-4 (6.3 ± 0.6 mg). Conversely, at DAI 15, the highest average root fresh weight was observed in the plants treated with BTL-M2 (33.12 ± 6.52 mg), which was significantly higher than the control plants where the lowest average root fresh weight was recorded at both DAI 10 (6.3 ± 1.8 mg) and DAI 15 (29.64 ± 7.36 mg; Figure 2C). The mean root dry weight (at 10 DAI) was recorded in BTL-M2 treated plants (1.39 ± 0.29 gm) followed by BRRh-4 (1.21 ± 0.60 gm) and untreated controls (0.26 ± 0.16 gm). Likewise, in the case of root dry weight, the highest average was recorded in the plants treated with BTL-M2 at DAI 15 (4.98 ± 0.97 gm) compared to that of BRRh-4 (4.43 ± 0.43 gm) and untreated control (3.01 ± 0.62 gm) plants (Figure 2D).

### *Paraburkholderia* and *Delftia* improve growth and grain yield of rice under nutrient deficient conditions

**Figure 3:**
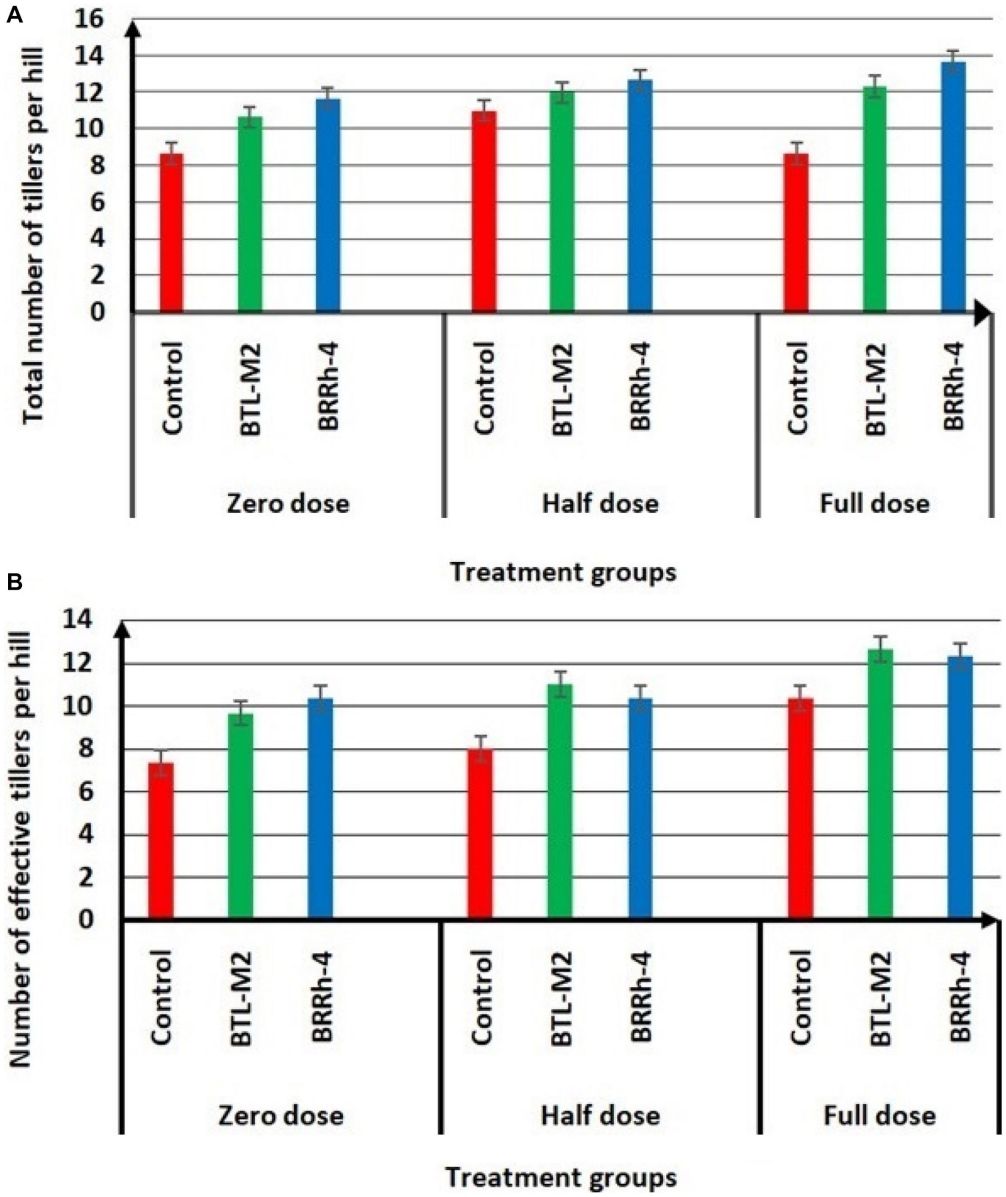
Effects of BTL-M2 and BRRh-4 on number of tillers per hill. **(A)** Effects of probiotic bacteria on total number of tillers per hill. **(B)** Effects of probiotic bacteria on number of effective tillers per hill. Zero dose: no chemical fertilizers used. Half dose: 50% of recommended N, P, and K fertilizers used. Full dose: 100% of recommended N, P, and K fertilizers used.

The application of BTL-M2 and BRRh-4 significantly increased growth and yield of rice. At the zero-dose of recommended N, P, and K fertilizers treatment, the plants with bacteria had a considerably higher growth compared to the rice plants treated with no bacteria (Figure 2). Application of both BTL-M2 and BRRh-4 increased plant heights irrespective of the fertilizer treatments. In the case 100% doses of chemical fertilizers, the average plant height obtained was 114.67 ± 1.66 cm, whereas, in the 50% doses of chemical fertilizer combined with the bacterial strains, BTL-M2 and BRRh-4, the average plant heights obtained were 115.33 ± 1.2 cm and 115.52 ± 1.45 cm, respectively. And these findings were statistically (*p* < 0.05, One-way ANOVA) almost similar to the 100% doses of chemical fertilizers application (Table 2). The total number of tillers per hill and the effective number of tillers per hill were also higher in rice plants treated with the bacteria. Applying 100% doses of chemical fertilizers, the total number of tillers per hill was 12.67 ± 0.33. On the other hand, the total number of tillers per hill in the combined application of 50% doses of chemical fertilizers with the bacterial strains, BTL-M2 and BRRh-4 gave statistically similar results (12.00 ± 0.99 and 12.91 ± 0.33, respectively; Figure 3A). Likewise, the number of effective tillers per hill in the combined application of 50% doses of chemical fertilizers with bacterial strains BTL-M2 and BRRh-4 were 9.83 ± 0.88 and 10.27 ± 0.66, respectively which statistically similar with the results obtained by the full doses of chemical fertilizers (10.33 ± 0.66; Figure 3B) only.

**Table 2:** Effects of probiotic bacteria on plant height of rice.

Treatments	Plant height (cm)		
	Zero dose	Half dose	Full dose
Control	91.67 ± 0.88^d^	106.00 ± 1.73^b^	114.67 ± 1.66^a^
BTL-M2	97.00 ± 1.73^c^	115.33 ± 1.2^a^	117.00 ± 0.57^a^
BRRh-4	98.22 ± 1.85^c^	115.52 ± 1.45^a^	116.88 ± 1.76^a^

Zero dose: no chemical fertilizers used. Half dose: 50% of recommended N, P, and K fertilizers used. Full dose: 100% of recommended N, P, and K fertilizers used. ^a,b,c,d^Superscripts in a row mean statistically significant difference (*p* < 0.05).

**Figure 4:**
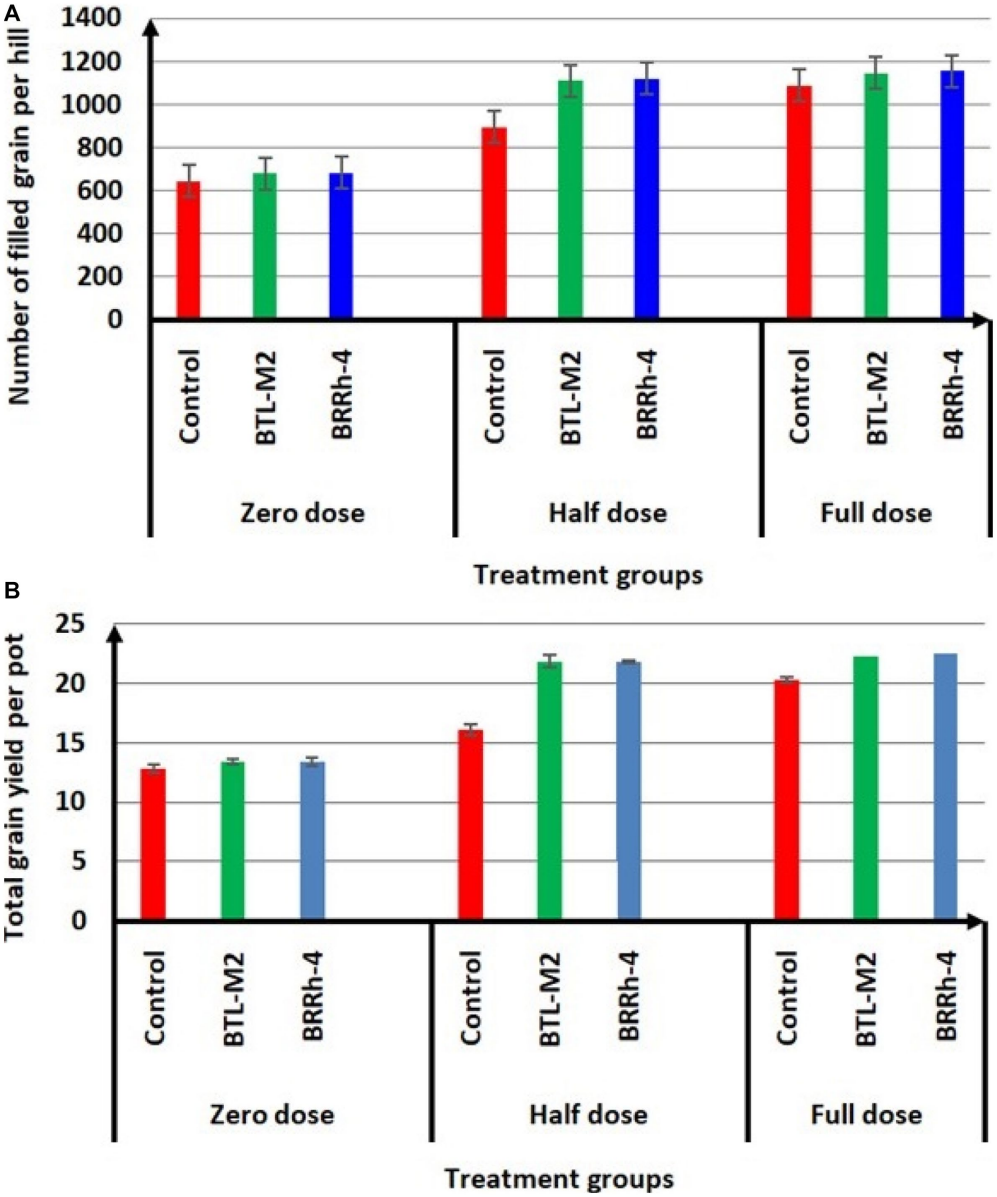
Effects of BTL-M2 (*Delftia* sp.; D) and BRRh-4 (*Paraburkholderia fungorum*; Pf) on rice grain yield. **(A)** Effects of probiotic bacteria on number of filled grain per hill. **(B)** Effects of probiotic bacteria on total grain yield of rice per pot. Zero dose: no chemical fertilizers used. Half dose: 50% of recommended N, P, and K fertilizers used. Full dose: 100% of recommended N, P, and K fertilizers used.

Application of probiotic bacteria significantly effects the filled and unfilled grain of rice. The maximum number of filled grain was recorded in the combination of 100% doses of chemical fertilizers plus BRRh-4 (1,155 ± 45.84) treatment followed by the same doses of chemical fertilizers combined with BTL-M2 (1,146 ± 26.21) and only by the treatment of 100% doses of chemical fertilizers (1,146 ± 26.21; Figure 4A). Interestingly, the number of filled grain in 50% recommended doses of chemical fertilizers in combination with BRRh-4 and BTL-M2 were 1,119 ± 13.45 and 1,113 ± 27.59, that were statistically similar to the 100% doses of chemical fertilizers (1,146 ± 26.21) without treatment of any bacteria. The lowest number of filled grain was obtained from 0% doses of chemical fertilizers (645 ± 32.56; Figure 4A) with no treatment of bacteria. In the 50% doses of chemical fertilizers, the average number of unfilled grains were significantly lower when combined with treatment of either probiotic bacterial strain BTL-M2 (256 ± 3.48) and BRRh-4 (253 ± 12.35), respectively compared to the rice treated with only chemical fertilizers (382 ± 42.55; Table 3). The application of the probiotic bacterial strains BRRh-4 or BTL-M2 alone reduced the number of unfilled grains of rice compared to untreated control. The 1,000-grain weight obtained from the 100% recommended full doses of chemical fertilizers was 19.24 ± 0.13 g whereas, the almost similar result (19.53 ± 0.22 g) was obtained from the 0% dose of chemical fertilizers when combined with the treatment of BRRh-4 (Table 4). The total grain yield per pot was higher in the 50% doses of chemical fertilizers treatment when combined with the treatment of BTL-M2 (21.98 ± 0.42 g) or BRRh-4 (21.64 ± 0.54 g) compared to the total grain yield per pot obtained by the treatment of rice with 100% recommended doses of chemical fertilizers (20.27 ± 0.12 g) but all these data were statistically similar (Figure 4B).

**Table 3:** Effects of probiotic bacteria on unfilled grain (nos.) per hill in pot.

Isolates	Unfilled grain		
	Zero dose	Half dose	Full dose
Control	176 ± 31.35^d^	382 ± 42.55^a^	214 ± 13.85^cd^
BTL-M2	322 ± 16.80^ab^	256 ± 3.48^bc^	194 ± 39.08^cd^
BRRh-4	239 ± 2.84^a^	253 ± 12.35^bcd^	254 ± 37.47^bcd^

Zero dose: no chemical fertilizers used. Half dose: 50% of recommended N, P, and K fertilizers used. Full dose: 100% of recommended N, P, and K fertilizers used. ^a,b,c,d^Superscripts in a row mean statistically significant difference (*p* < 0.05).

**Table 4:** Effects of probiotic bacteria on 1,000 grain weight of rice plant in pot.

Isolates	1,000 grain weight (g)		
	Zero dose	Half dose	Full dose
Control	18.61 ± 0.37^abc^	17.95 ± 0.22^c^	19.24 ± 0.13^ab^
BTL-M2	18.01 ± 0.21^bc^	19.25 ± 0.43^ab^	19.69 ± 0.37^ab^
BRRh-4	19.53 ± 0.22^a^	19.53 ± 0.54^ab^	19.69 ± 0.23^ab^

Zero dose: no chemical fertilizers used. Half dose: 50% of recommended N, P, and K fertilizers used. Full dose: 100% of recommended N, P, and K fertilizers used. ^a,b,c,d^Superscripts in a row mean statistically significant difference (*p* < 0.05).

Plant growth-promoting rhizobacteria are associated with plant roots and increase plant productivity and resistance to abiotic stresses. In the current study, the application of probiotic bacteria, *P. fungorum* BRRh-4 and *Delftia* sp. BTL-M2 remarkably improved the growth and yield of rice. Interestingly, both the probiotic bacteria with 50% recommended doses of the N, P and K fertilizers resulted in almost equivalent growth and yield of rice when treated with 100% doses of these fertilizers. Our results clearly indicate that application of BRRh-4 and BTL-M2 strains could reduce the requirements for 50% N, P, and K fertilizers in rice production. Moreover, the continuous application of synthetic fertilizers (*CF*) has adversely affected the natural environment.

### Probiotic bacteria application improves diversity of bacteria in root and rhizosphere soils of rice

#### Structure and composition of bacteriome

**Figure 5:**
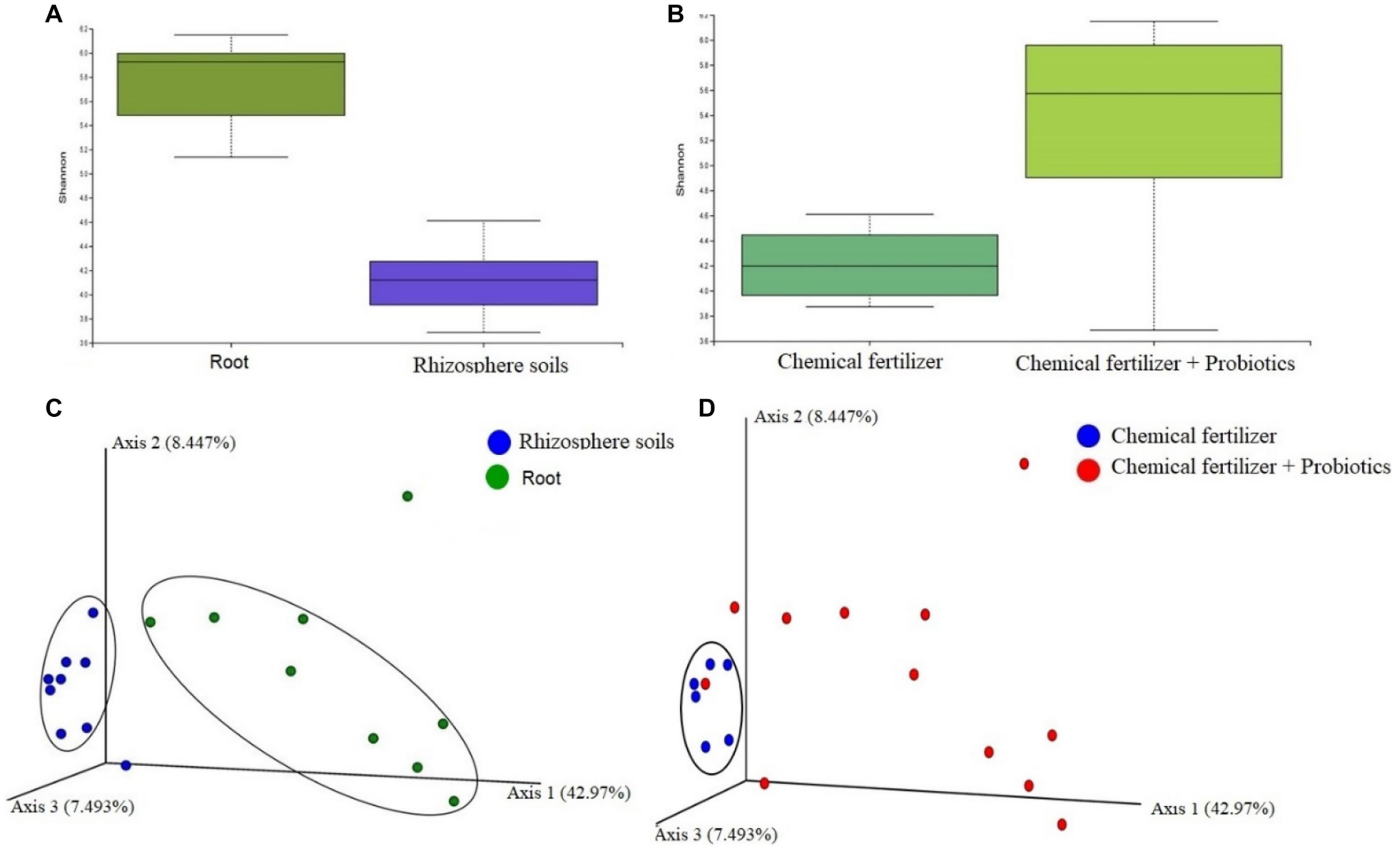
Microbiome diversity in root and rhizosphere soils, and chemical fertilizer (*CF*) and *CF* + probiotics treated metagenomes (at genus level). **(A)** Microbial alpha-diversity calculation with Shannon diversity index in **(A)** root and rhizosphere soils, and **(B)** *CF* and *CF* + probiotics treated metagenomes. The Shannon diversity remained significantly higher in root (PERMANOVA, *p* = 0.00035) and *CF* + probiotics treated (PERMANOVA, *p* = 0.0312) metagenomes compared to rhizosphere soils and *CF* metagenomes, respectively. **(C)** The PCA plot (beta diversity) at the genus level revealed that most of the rhizosphere soils related samples (blue circles) clustered closely (encircled by black ellipse) while the samples of root samples (green circles) were distributed distantly showing the significant microbial disparity between the metagenomes. **(D)** The genus-level PCA plot revealed that most of the *CF* treated samples (blue circles) clustered closely (encircled by black ellipse) while the samples of *CF* treated samples (red circles) were distributed diversely showing the significant microbial disparity between the metagenomes.

To see whether application of probiotic bacteria effect on the population and diversity of bacteriome in rhizosphere soils and the roots, we conducted a metagenomics analysis. The ribosomal (16S rRNA) gene amplicon sequencing yielded a total of 4,379,162 reads with an average number of 243,286.78 reads per sample and an average GC content of 50.81% (Data S1). A total of 2,039 unique Operational Taxonomic Units (OTUs) were identified with an average number of 228.28 OTUs per sample (maximum = 608, minimum = 60). The samples of root bacteriome always possessed a higher number of OTUs per sample compared to that of the bacteriome of rhizosphere soils (Supplementary Data S1). We found significant differences (PERMANOVA, *p* = 0.00035) in alpha-diversity (Shannon estimated) between the root (R) and rhizosphere soil (S) metagenomes, showing higher diversity in the microbial ecosystem of root samples (Figure 5A). We compared the distribution of microbial community in chemical fertilizer (*CF*), *CF* + BTL-M2, and *CF* + BRRh-4 treated samples which revealed that the within-sample (alpha) diversity remained significantly higher (PERMANOVA, *p* = 0.0312) in the samples of combined *CF* and probiotics treated metagenomes than that of only *CF* treated group (Figure 5B). The principal component analysis (PCA) also showed significant microbial disparity (PERMANOVA, *p* = 0.002) among root and rhizosphere soils (Figure 5C), and *CF*, *CF* + BTL-M2, and *CF* + BRRh-4 treated metagenomes (Figure 5D).

**Figure 6:**
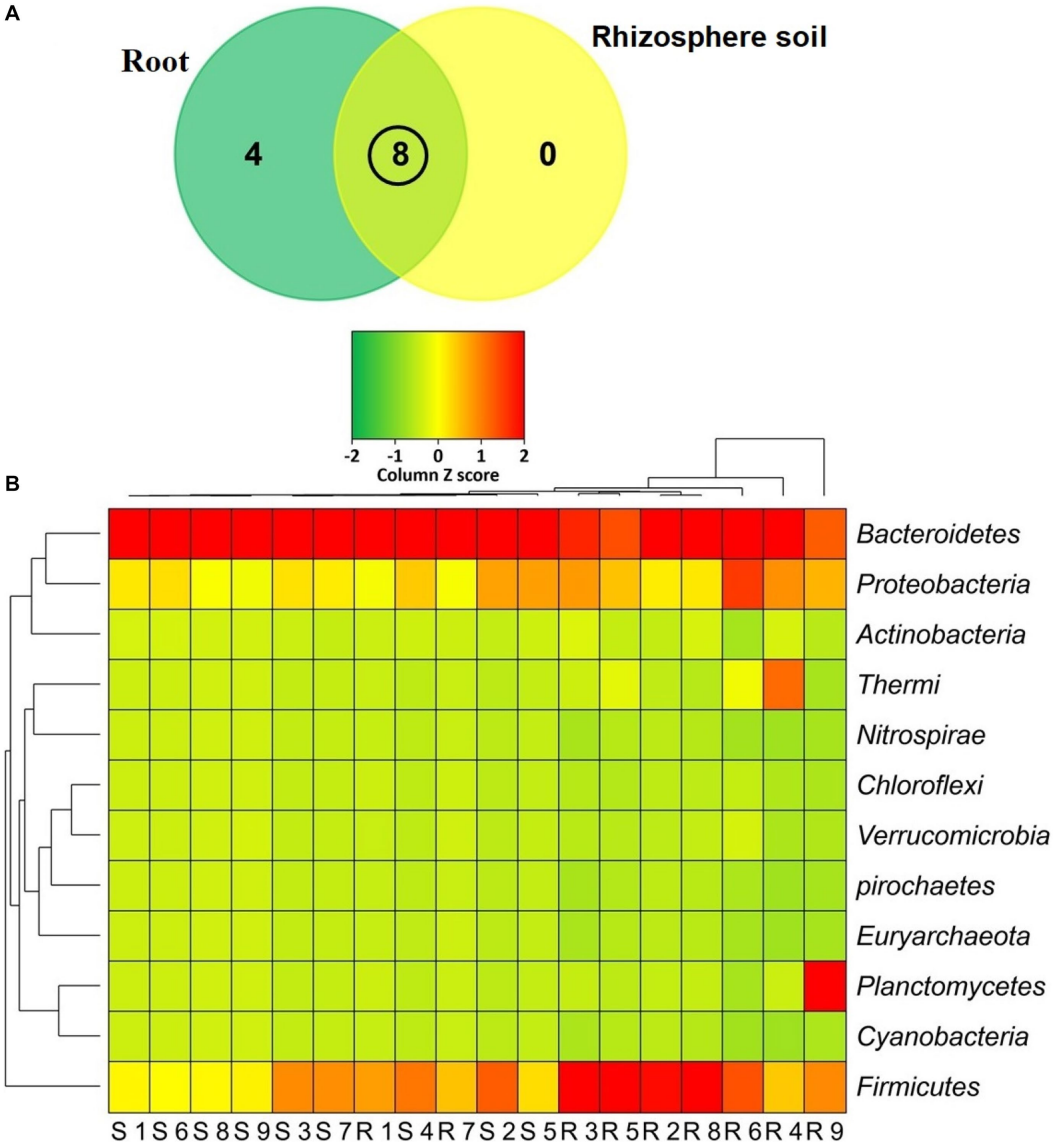
Taxonomic composition of the microbiome at phylum level. **(A)** Venn diagram representing the unique and shared bacterial phyla detected in rhizosphere soil (S1-S9) and root (R1-R9) samples. A total of 12 phyla were detected in both metagenomes where 8 phyla were found to be shared. **(B)** Heat map showing the composition and relative abundance of the detected phyla. The less abundant phyla in a given sample are shown in green, those that had medium abundant are in yellow, and phyla that are more abundant are represented in red. The varying color codes (Z-score) indicate the presence and completeness of each phylum, expressed as a value between −2 (low abundance) and 2 (high abundance).

**Figure 7:**
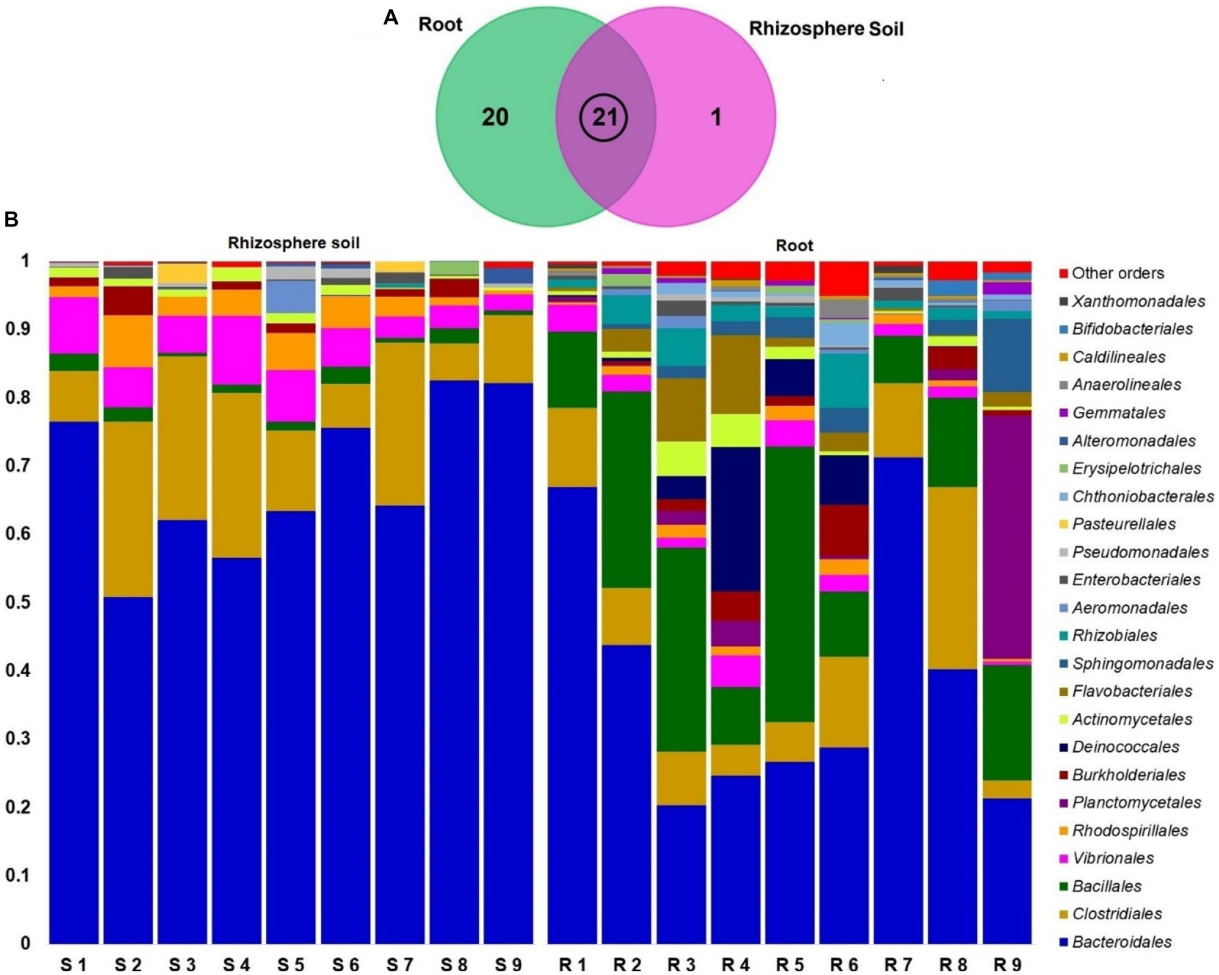
Taxonomic composition of root and soil microbiome at order level. **(A)** Venn diagram showing unique and shared bacterial orders in rhizosphere soil (S1-S9) and root (R1-R9) metagenomes. The microbiome sharing between the conditions is indicated by the black circle. **(B)** The relative abundances of the 24 most abundant orders of bacteria are sorted by ascending patterns, with the remaining orders represented by ‘Other orders’. Each stacked bar plot represents the abundance of bacteria in each sample of the corresponding category.

The classified sequences were aligned to 12 bacterial phyla in both root and rhizosphere soil samples (Supplementary Table S1), of which 66.67% (8/12) phyla were shared between the metagenomes, and the root (R) metagenome had a unique association of 33.33% (4/12) phyla (Figure 6A). The root (R) metagenome was mainly composed of *Bacteroidetes* (42.91%), *Firmicutes* (29.03%), *Proteobacteria* (13.51%), *Planctomycetes* (5.78%), *Thermi* (4.15%), *Actinobacteria* (2.01%) and *Verrucomicrobia* (1.07%; contributing to ~99.0% of the total sequences, Kruskal Wallis test, *p* = 0.021), and the rest of the detected phyla had a relatively lower abundance (< 1.0%; Figure 6B). On the other hand, the rice rhizosphere was mostly comprised of *Bacteroidetes* (67.25%), *Firmicutes* (17.32%), and *Proteobacteria* (14.19%; Figure 6B; Supplementary Table S1). Moreover, we detected 42 bacterial orders in both root and soil metagenomes (41and 21 orders in R and S metagenomes, respectively), of which 50% of the orders was shared between the conditions (Figure 7A; Supplementary Table S2). *Bacteroidales* (67.25%) was found as the single most predominant bacterial order detected in soil metagenome, however, other abundant orders in this metagenome were *Clostridiales* (15.47%), *Vibrionales* (6.27%), *Rhodospirillales* (3.53%), *Bacillales* (1.54%), *Burkholderiales* (1.37%) and *Actinomycetales* (1.12%; Figure 7B; Supplementary Table S2). The predominant bacterial orders in rice root metagenome were *Bacteroidales* (39.60%), *Bacillales* (19.19%), *Clostridiales* (9.44%), *Planctomycetales* (5.21%), *Deinococcales* (4.15%), *Flavobacteriales* (3.16), *Sphingomonadales* (2.70%), *Vibrionales* (2.56%), *Rhizobiales* (2.51%), *Burkholderiales* (1.80%), *Actinomycetales* (1.68%) and *Rhodospirillales* (1.34%), and rest of the orders had a relatively lower abundance (<1.0%; Figure 7B; Supplementary Table S2). Similar levels of bacterial diversity in rice root and rhizosphere soil have been reported elsewhere. However, this is the first report on the modulation of bacterial diversity in the rice roots and rhizosphere soils due to the application of BRRh-4 and BTL-M2 with varying doses of N, P, and K fertilizers.

### Microbial community of rice roots differs from rhizosphere soils

**Figure 8:**
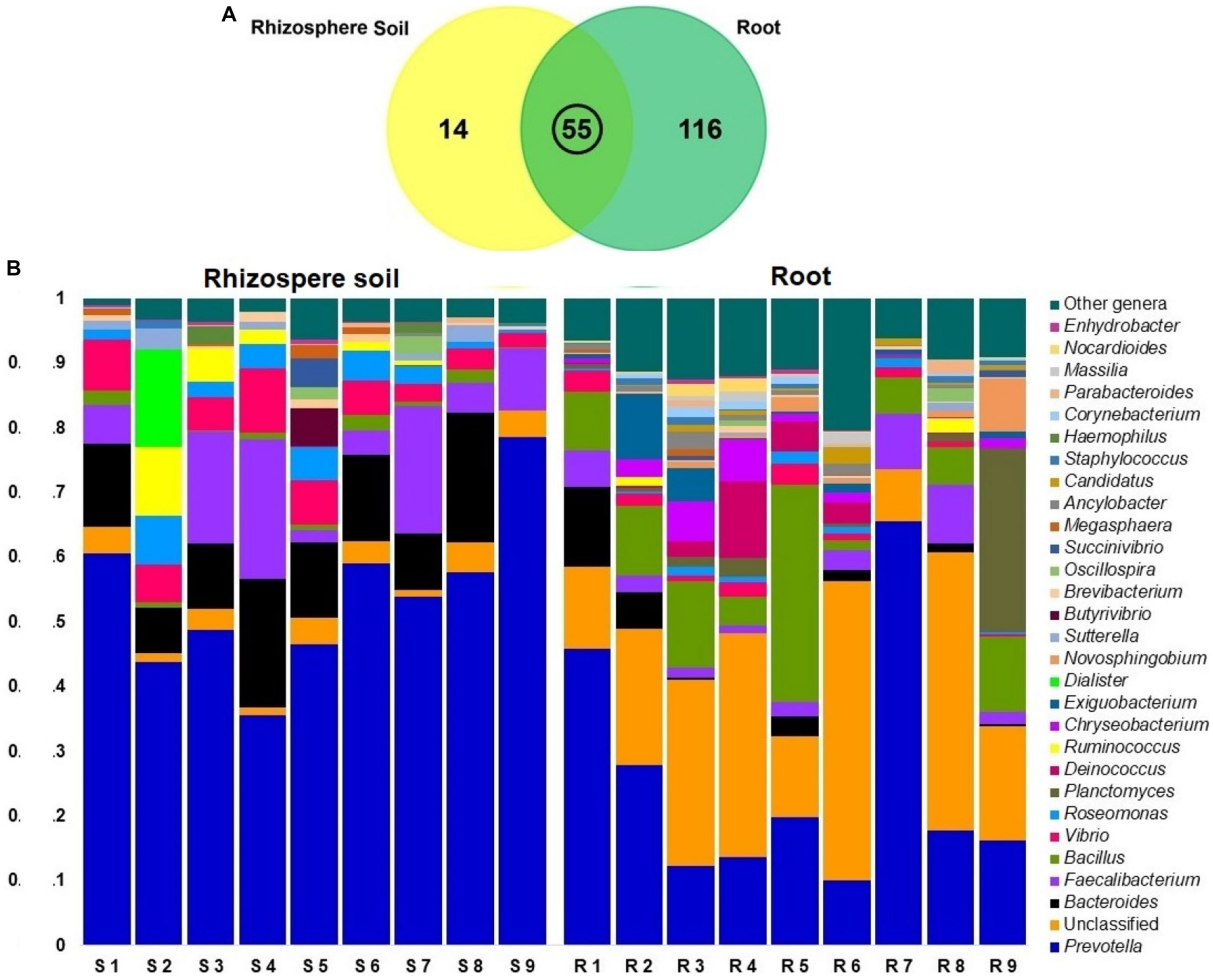
Taxonomic composition of root and soil microbiome at genus level; **(A)** Venn diagram showing unique and shared bacterial genera in rhizosphere soil and root metagenomes. **(B)** Taxonomic composition of the microbiome at genus level in rhizosphere soil (S1-S9) and root (R1-R9) samples. The relative abundance of top abundant 29 genera of bacteria are sorted by ascending order, with the remaining genera represented by ‘Other genera’. Each stacked bar plot represents the abundance of bacteria in each sample of the corresponding category.

In contrast to rice rhizosphere soils communities, bacterial communities in root showed significantly high diversity (*p* = 0.019, Kruskal Wallis test). At the genus level, we obtained a total of 185 bacterial genera from both the root and rhizosphere soil metagenomes, of which 171 and 69 genera were detected in root and rhizosphere soils. The unique bacterial genera found in both rice root and rhizosphere soil metagenomes were 62.70% (116/185) and 7.57% (14/185), respectively. However, 29.7% (55/185) genera were shared between these two metagenomes (Figure 8A; Supplementary Data S1). Even though, *Prevotella* was found as the predominant genera in both metagenomes, relative abundance of this genus remained two-fold higher in rhizosphere soil metagenome (52.02%) than the rice roots (25.04%; Supplementary Table S3). The other predominant bacterial genera detected in rhizosphere soil metagenome were *Bacteroides* (12.38%), *Faecalibacterium* (9.50%), *Vibrio* (5.94%), *Roseomonas* (3.40%), *Delftia* (3.02%), *Ruminococcus* (1.76%), *Bacillus* (1.20%), *Dialister* (1.16%), *Butyrivibrio* (107%). And rest of the genera had a relatively lower abundance (<1.0%; Figure 8B; Supplementary Data S1). Conversely, *Bacillus* (11.07%), *Planctomyces* (4.06%), *Faecalibacterium* (3.91%), *Deinococcus* (2.97%), *Bacteroides* (2.61%), *Chryseobacterium* (2.30%), *Exiguobacterium* (1.91%), *Vibrio* (1.78%), and *Novosphingobium* (1.60%) were the most abundant bacterial genera in rice roots. The rest of the genera had a lower relative abundance (<1.0%; Supplementary Table S3). Surprisingly, the root metagenome had many-fold higher unclassified bacterial genera (24.86%) than the soil metagenome (3.01%; Figure 8B; Supplementary Data S1).

### Application of probiotic bacteria remarkably changes the rice associated bacterial community

**Figure 9:**
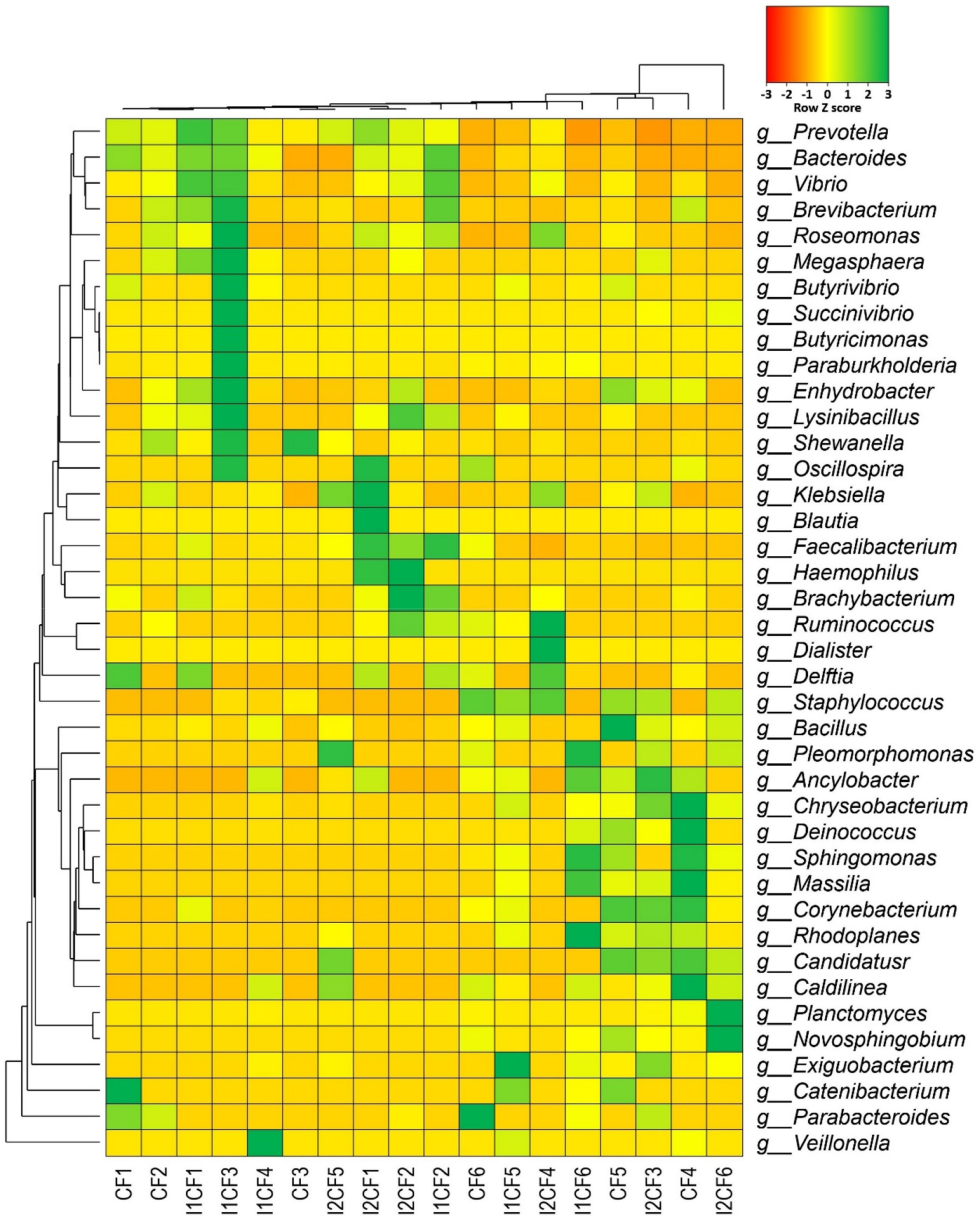
Taxonomic composition of the microbiome in different treatment groups (at genus level). Heatmap showing the composition and relative abundance of the top abundant 40 bacterial genera in the metagenomes of chemical fertilizer (CF1-CF6), *CF* + *Delftia* (I1CF1-I1CF6), and *CF* + *Paraburkholderia* (I2CF1-I2CF6). Here: CF1 – CF2: zero dose chemical fertilizer; CF3 – CF4: half dose chemical fertilizer; CF5 – CF6: full dose chemical fertilizer; I1CF1 – I1CF2: *Delftia* + zero dose chemical fertilizer; I1CF3 – I1CF4: *Delftia* + half dose chemical fertilizer; I1CF5 – I1CF6: *Delftia* + full dose chemical fertilizer; I2CF1 – I2CF2: *Paraburkholderia* + zero dose chemical fertilizer; I2CF3 – I2CF4: *Paraburkholderia* + half dose chemical fertilizer; I2CF5 – I2CF6: *Paraburkholderia* + full dose chemical fertilizer. Zero dose: no chemical fertilizers used. Half dose: 50% of recommended N, P, and K fertilizers used. Full dose: 100% of recommended N, P, and K fertilizers used. The less abundant generated by a given samples are shown in red (Z score = −3), those having medium abundance are presented in yellow (Z score = 0), and genera that are more abundant represented in green (Z score = 3).

The most evident differences between *CF* and *CF* + probiotics (*CF* + BTL-M2 and *CF* + BRRh-4) treatments were detected in the fraction of reads assigned to a genus. The microbial community in the treated rice metagenomes varied significantly (*p* = 0.002, Kruskal Wallis test) showing the potential efficacy of combined *CF* and probiotics therapy over sole application of *CF.* Of the detected bacterial genera (n = 185), 133 and 161 genera were detected in *CF*, and *CF* + probiotic bacteria treated rice groups, respectively (Supplementary Figure S3A). Therefore, treatment of rice by a combination of *CF* and probiotic bacteria had the inclusion of 52 (28.11%) bacterial genera (Supplementary Figure S3A). Of them, *Planctomyces* (2.98%), *Chryseobacterium* (1.68%), *Exiguobacterium* (1.40%), *Novosphingobium* (1.14%) and *Ancylobacter* (0.74%) were the most abundant genera (<0.5%; Supplementary Data S1). Although, *Prevotella* remained as the most abundant bacterial genus (47.19%) in both *CF* and *CF* + probiotics metagenomes, the relative abundance of this genus was found higher in *CF* treated samples (49.35%) than the *CF* + probiotics (34.26%) sample groups. The relative abundance of the rest of the bacterial genera also varied significantly (p = 0.002, Kruskal Wallis test) in both *CF* and *CF* + probiotics treated samples with predominantly unclassified bacterial genera in *CF* + probiotics samples (18.86%; Figure 9; Supplementary Data S1). The composition of microbial community and their associated relative abundances also varied significantly (*p* = 0.044, Kruskal Wallis test) within *CF* + probiotics treated samples keeping higher number of bacterial genera (153) in *CF* + BRRh-4 treated samples than *CF* + BTL-M2 treated samples (n = 120; Supplementary Figure S3B). However, out of 175 genera detected in both metagenomes, 56% (98/175) genera were found to be shared (Supplementary Figure S3A; Supplementary Data S1). The *CF* + BRRh-4 treated samples were therefore enriched with higher percentage (31.42%) of unique bacterial genera compared to *CF* + BTL-M2 treated metagenomes (12.57%; Supplementary Figure S3B; Supplementary Data S1).

Considering the divergence of microbiome composition across all of the sample groups (*CF*, *CF* + BTL-M2 and *CF* + BRRh-4; Supplementary Figure S4A), we found that 20.54% (38/185) bacterial genera were shared among all of the three metagenomes (Supplementary Figure S4B; Supplementary Data S1). These shared bacteriome were mostly represented by *Prevotella* (23.23 to 49.35%), *Bacteroides* (1.11 to 12.77%), *Faecalibacterium* (4.13 to 8.83%), *Vibrio* (1.66 to 7.12%), *Roseomonas* (0.87 to 3.95%; Figure 9; Supplementary Data S1). Moreover, in this metagenomic study, we also found significant effect of fertilizer dosage on the composition and diversity of the associated microbiomes in different treatment groups. For instance, we detected 25 bacterial genera in CF1 and CF2 with zero dose chemical fertilizer whereas 56 and 69 bacterial genera were detected in CF3 – CF4 and CF5 – CF6 after application of half and full doses of chemical fertilizer, respectively. Similarly, we detected 26 bacterial genera in I1CF1 and I1CF2 with BTL-M2+ zero dose chemical fertilizer while 45 and 65 bacterial genera were detected in I1CF3 – I1CF4 and I1CF5 – I1CF6 after application of BTL-M2+ half dose and BTL-M2+ full dose of chemical fertilizer, respectively. Correspondingly, 29, 51 and 50 genera were detected in I2CF1 – I2CF2, I2CF3 – I2CF4 and I2CF5 – I2CF6 after application of BRRh-4+ zero dose, BRRh-4+ half dose, BRRh-4+ full dose of chemical fertilizer (Figure 9; Supplementary Data S1).

In addition, microbiome signature of rice was significantly modulated (inclusion or depletion) by the application of the probiotic bacteria with or without chemical fertilizers. Interestingly, treatment specific association of some genera was traced in the samples of rice root and rhizosphere (Table 5), and some of the genera were absent in the samples of respective treatment. For example, abundance of the genera *Deinococcus*, *Chryseobacterium*, *Nocardioides, Planctomyces, Ancylobacter, Exiguobacterium* etc. were found in the treatments of rice with any of the probiotic bacteria with or without fertilizers. Interestingly, *Sphingomonas*, *Massilia* and *Novosphingobium* were only found associated with BRRh-4 treatment. On the other hand, abundance of *Brevibacterium* was recorded in rice irrespective of the treatments. Conversely, *Ruminococcus* and *Dialister* were found to be colonized in the root and rhizosphere soils of rice plant that received *CF* treatment (at both zero and half of the recommended dose), however, diminished after probiotic bacterial application along with *CF* [i.e., in *CF* + BTL-M2 (I_1_CF_0_ and I_1_CF_0.5_) and *CF* + BRRh-4 (I_2_CF_0_ and I_2_CF_0.5_)] (Table 5). Conversely, *Planctomyces*, *Ancylobacter*, *Clostridium* and *Anaerolinea* were not detected in samples that received only *CF* treatments, however, there was a recruitment and/or inclusion of these two genera after probiotic bacterial application along with *CF* at both zero and half dose. Surprisingly, probiotic bacteria *Delftia* and *Paraburkholderia* treatment coupled with *CF* at half of their recommended dose depleted the *Brachybacterium* from both rice root and rhizosphere soil samples (Table 5).

**Table 5:** Treatment associated changes in bacterial community at genus-level (top 35 genera).

Microbiota	Treatment groups					
	CF_0_	CF_0.5_	I_1_CF_0_	I_1_CF_0.5_	I_2_CF_0_	I_2_CF_0.5_
*Prevotella*						
*Faecalibacterium*						
*Bacteroides*						
*Bacillus*						
*Vibrio*						
*Roseomonas*						
*Deinococcus*	X		X			
*Ruminococcus*			X	X	X	X
*Dialister*			X	X	X	X
*Sutterella*				X		
*Chryseobacterium*	X	X	X			
*Brevibacterium*						
*Oscillospira*	X	X		X		X
*Haemophilus*	X				X	X
*Planctomyces*	X	X				
*Ancylobacter*	X	X				
*Blautia*	X			X		
*Candidatus*	X		X			
*Massilia*	X	X	X	X		
*Novosphingobium*	X	X	X	X		
*Catenibacterium*	X			X		
*Exiguobacterium*	X	X	X			
*Nocardioides*	X	X	X			
*Corynebacterium*		X	X			
*Megasphaera*			X		X	
*Enhydrobacter*						
*Sporomusa*	X	X	X			
*Methylosinus*	X	X	X			
*Streptococcus*						X
*Klebsiella*	X	X		X	X	X
*Staphylococcus*		X	X			X
*Sphingomonas*	X	X	X	X		
*Brachybacterium*				X		X
*Clostridium*	X	X				
*Anaerolinea*	X	X				

Here: CF_0_: Zero dose chemical fertilizer; CF_0.5_: Half dose chemical fertilizer control; I_1_CF_0_: BTL-M2 + zero dose chemical fertilizer; I_1_CF_0.5_: BTL-M2 + Half dose chemical fertilizer; I_2_CF_0_: BRRh-4+ zero dose chemical fertilizer; I_2_CF_0.5_: BRRh-4+ Half dose chemical fertilizer. X: bacterial genus absents in respective group(s).

## Discussion

The use of plant probiotic bacteria (PPB) with minimum or no chemical fertilizers is considered as an eco-friendly means to carry out intensified rice production without degrading soil health and environment. Over recent decades, attention in the application of PPB in rice production has increased dramatically due to their ability to act as plant-growth regulators (Harman and Uphoff, 2019). Crop plants harbour and rely on rhizosphere-associated microbiomes for nutrition, which is crucial to their productivity. The excessive use of nitrogenous, phosphorous and potash fertilization is currently one of the main problems affecting soil health, eutrophication and high input cost of rice production in intensive agriculture system (Ha et al., 2015; Dong et al., 2021). In this study, we demonstrated that application of two rice probiotic bacteria, *P. fungorum* strain BRRh-4 and *Delftia* strain BTL-M2 produce statistically equivalent or higher grain yield of rice with 50% reduced doses of recommended N, P, and K fertilizers compared to 100% doses of these fertilizers without the application of any probiotic bacteria. Furthermore, using metagenomics analysis, we demonstrated that application of probiotic bacteria significantly modulates diversity and population of bacteriome in roots and rhizosphere of the treated rice plants. Interestingly, we found the recruitment or abundance of the specific genera of bacteria only in the probiotic treated rice roots and rhizosphere soils (Table 5). Although some plant growth promoting traits of *Delftia* sp. and *Paraburkholderia* sp. have been reported (Chaudhary et al., 2012; Ha et al., 2015), this study for the first time demonstrated the application of these rice probiotic bacteria to improve growth and yield of rice likely through modulating and/or improving the diversity of the native bacteriome of rice and rhizosphere soils.

In this study, we found that seed germination rate was higher in both *Delftia* strain BTL-M2 (89%) and *P. fungorum* strain BRRh-4 (88%) treated rice compared to untreated controls (77%). Previous studies have reported a number of endophytic bacteria with high nitrogen fixing enzyme activity and probiotic potential that were isolated from rice (Chaudhary et al., 2012; Khan et al., 2017). Enhancement of the rice seed germination rates, seedling growth and yield of rice in nutrient deficient soils by probiotic bacteria have also been reported in a recent report (Khan et al., 2017). A previous study has reported that both BTL-M2 (89%) and BRRh-4 strains produced phytohormone IAA and solubilize phosphorus *in vitro* (Khan et al., 2017). The enhanced root and shoot growth in rice seedlings might be linked with the production of IAA and phosphorus solubilization ability of the bacterial strains. An association of phytohormone production with root and shoot growth promotion by probiotic bacteria have been reported (Khan et al., 2017; Méndez-Gómez et al., 2021). Several studies have reported that plant probiotic bacteria influence plant biomass production by the production of phytohormone, solubilization of phosphorus and some other mechanisms (Hu et al., 2021). The increased shoot weight of rice seedlings could be linked with the production of IAA, fixation of atmospheric nitrogen and solubilization of essential nutrient elements in soils by the bacterial strain BRRh-4 and BTL-M2 (Sharma and Shukla, 2021).

Several lines of evidence suggest that plant-associated *P. fungorum* and *Delftia* sp. promote growth and dry matter production by various mode of actions (Singh et al., 2021). Supplementing synthetic fertilizers partially with the plant-growth-promoting probiotic bacteria is an alternate option to reduce chemical fertilizer (*CF*) usage in crop production (Singh et al., 2021). One of the interesting findings of our current study is that application of both *Delftia* strain BTL-M2 and *P. fungorum* strain BRRh-4 with 50% recommended doses of N, P, and K fertilizers produced almost equivalent or higher yield of rice compared to the application of 100% N, P, and K fertilizers only. Our results suggest that application of these probiotic bacteria as biofertilizer could reduce 50% requirement of chemical fertilizers (*CF*) in rice. In earlier studies, we found that both *Paraburkholderia* and *Delftia* solubilize minerals, phosphates and potassium, fix atmospheric nitrogen and produce IAA *in vitro*. The presence of the applied bacterial population and modulated population and diversity of the native bacteriome shown in this might be involved in growth and yield promotion of rice (Khan et al., 2017; Rahman et al., 2018a). Various plant growth promoting traits including fixation of atmospheric nitrogen by both *Paraburkholderia* and *Delftia* spp. separately have been reported (Trân Van et al., 2000; Han et al., 2005; Rahman et al., 2018a). Kobua et al. (2021) previously demonstrated that the combined application of *CF* + plant growth promoting rhizobacteria (PGPR) improved the growth and yield of rice. Conversely, Nguyen et al. (2019) and Akram et al. (2013) reported that the tiller number of monocot plants such as rice was less (significantly) affected by *CF* + probiotic bacteria treatments than the crop height and other vegetative parameters. In fact, plants treated with some elite strains of probiotic bacteria form resilient cell structures and have better metabolic processes along the culm area (Parra-Cota et al., 2014; Kobua et al., 2021). However, the results obtained in the current study in pot culture condition are needed to be confirmed by field experiment. A large body of the literature indicate that the application of probiotic bacteria enhance the growth and yield of crop by both direct and indirect mechanisms including phytohormone production, nutrient acquisition, inhibiting the growth of pathogenic fungi and also inducing systemic resistance in the host plants (Khan et al., 2016; Hu et al., 2021; Méndez-Gómez et al., 2021). Our study demonstrated that two rice probiotic bacteria with various plant growth promoting traits are potential candidates for the development of biofertilizers to reduce the cost of rice production and improve the soil health. A further large field evaluation of these bacteria alone or in combination is needed before recommending them as biofertilizers for rice.

Interestingly, we found that our applied bacterial population significantly low in the rhizosphere soils and roots but a remarkable change of the diversity and population structure occurred in other bacteriome in both rhizosphere and roots due to their application. We assume that the modulation in bacteriome could be due to the interactions and/or cross-talk of the applied bacteria with the native bacteria in rice rhizosphere that ultimately resulted in the higher yield of rice. A further study is warranted to clarify the functional aspects of the changed bacteriome that impacted to the growth and yield of rice (Khan et al., 2017). Microbial communities in the rhizosphere play a key role in solubilisation and availability of essential nutrient elements to the plants and thus influence plant health, growth and yield (Friesen et al., 2011). In turn, rhizosphere soil microorganisms heavily rely on root exudates, such as carbon metabolites and other nutrients, as growth substrates. Plant species directly influence soil microbial communities through these root exudates which change as the plant matures (Berg and Smalla, 2009). A number of studies have shown that microbial taxa identified through the metagenomic analysis are all widely present in soil, including species from the *Actinobacteria*, *Chloroflexi*, *Planctomycetes*, *Proteobacteria*, and *Verrucomicrobia* phyla (Berg and Smalla, 2009; Zhang and Lv, 2020). Our study also reported the rice associated bacteria cultivated in the soils of Bangladesh under the treatment of probiotic bacteria with or without the application of major chemical fertilizers used in rice cultivation. The beneficial effects of the application of probiotic bacteria on rice and many other crops have been reported (Yanni et al., 2001; Yadav et al., 2020; Ngalimat et al., 2021). For example, soil bacteria such as *Pseudomonas*, *Bacillus* and *Rhizobium* increase the concentration of plant available phosphorus (P) in soil by releasing phosphatase enzymes or organic chelates (Lazcano et al., 2021). Additionally, certain strains of *Bacillus*, *Pseudomonas*, *Paraburkholderia* and *Acinetobacter* are thought to increase the availability and uptake of micronutrients such as Zn and Fe for mung bean, rice and corn (Goteti et al., 2013; Lazcano et al., 2021).

The plant microbiome has potential for improving crop productivity and sustainability (Kim and Lee, 2020). In this study, we characterized the bacteria communities associated with rice roots and rice rhizosphere soils after the application of rice probiotic bacteria, BRRh-4 and BTL-M2 with varying doses of chemical fertilizers. We detected 133 and 161 genera in *CF* and *CF* + probiotic bacteria treated rice groups, respectively (Supplementary Figure S3A). Some of these genera such as *Prevotella* were abundant in rice root samples and other genera were more abundant with rhizosphere soils. *Prevotella* species have been isolated from human and animal gut samples, and also from root and soil samples of the irrigated rice (Ueki et al., 2007; Tett et al., 2021). This genus is also detected in the rumen of the ruminant as high abundance after feeding rice straw supplemented with nitrogenous substances (Latham et al., 2018). *Prevotella* species are found to play a significant role in breakdown of carbohydrate in the gut of animals (Precup and Vodnar, 2019). Furthermore, *P. paldividence* was isolated as hemicellulose decomposing bacteria from plant residue and rice roots in irrigated rice-field soils (Ueki et al., 2007). Therefore, the high abundance of *Prevotella* in our irrigated rice root and rhizosphere might be partly linked with the cow dung application in the pot soils. The genus *Prevotella* plays major roles in carbon, carbohydrate and nitrogen metabolism (Kim et al., 2017). In term of ‘One Health Concept’, interlinking of different ecosystems by *Prevotella* is one of the best examples of soil-water-plant–animal-human continuum.

In the present study, we recovered several unique bacterial taxa of rice roots belonging to the genera of *Chryseobacterium*, *Exiguobacterium*, *Novosphingobium*, *Massilia*, *Nocardioides*, *Methylosinus*, *Streptomyces*, *Sphingomonas*, and *Paenibacillus*. Many of these genera have shown *in vitro* and/or in planta beneficial effects on promoting the growth, productivity and/or improve disease resistance of plants (Zhou et al., 2020). For instance, the beneficial probiotic bacteria of *Chryseobacterium* genus can inhibit pathogens, and modulate other beneficial microbes (Jinendiran et al., 2019), and many strains of *Exiguobacterium* are known as potential sources for acquisition and fixation of nutrients (Pandey, 2020). *Novosphingobium*, a novel rhizosphere-associated bacterium with plant beneficial properties isolated from saline-tolerant pokkali rice (Krishnan et al., 2017). The probiotic bacteria *Sphingomonas* increases plant growth rate and alters the rhizosphere microbial community structure of *Arabidopsis thaliana* under drought stress (Luo et al., 2019). The Gram-positive rhizosphere bacterium, *Paenibacillus* promotes plant growth and produces various antibiotics (Jeong et al., 2019). Discussing the functional characters of an endophytic community colonizing rice roots, del Barrio-Duque et al. (2020) speculated that the endophytic community of rice play a vital role in plant growth promotion, improvement of stress tolerance, biocontrol pathogenic microorganisms, and bioremediation (del Barrio-Duque et al., 2020).

Our study catalogued the bacteriome in rice roots and rhizosphere, and demonstrated how the application of rice probiotic *Paraburkholderia* and *Delftia* with or without application of chemical fertilizers modulate the bacterial community in both roots and rhizosphere soils. Our results indicate that bacterial populations are highly influenced by the application of *CF* alone and/or in combination with probiotic bacteria. Interestingly, combined application *CF* and probiotic bacteria had the highest bacterial diversity. Furthermore, this higher diversity of bacteria in root and rhizosphere of rice was correlated with the higher growth and grain yield. These results suggest that manipulation of bacterial community associated with rice by the application of probiotic bacteria could be an alternative and sustainable approach for reduction of the dependency of synthetic chemical fertilizers in rice production. Obviously, the enhancement of microbial diversity by the application of probiotic bacteria *Paraburkholderia* and *Delftia* sp. might improve the soil health.

A hallmark of our research findings is that we found the abundance or recruitment of specific genera of known plant growth promoting bacteria in rice by the application of rice probiotic bacteria, *Paraburkholderia* and *Delftia*. The abundance/presence of *Sphingomonas*, *Novosphingomonas* and *Massilia* only with the treatment of rice with a strain of *Paraburkholderia* (BRRh-4) was highly interesting and merit further investigation for understanding the mechanism involved. Our results indicate that the growth promotion and yield increment of rice were not only achieved by the effects of the applied probiotic bacteria but also largely by the effects of the modulated bacteriome and other unknown microbiome.

In the current study, probiotic bacteria application significantly modulates the structures, diversity and population of the rhizosphere bacteria of rice. These modulations might be linked with the significant growth-promotion and grain yield increase in rice by the application of BRRh-4 and BTL-M2. Production of phytohormones, solubilisation of essential nutrient elements (e.g., P and K) and fixation of atmospheric nitrogen in soils by the *Paraburkholderia* and *Delftia* spp. have been reported (Trân Van et al., 2000; Han et al., 2005; Rahman et al., 2018a). A further shotgun metagenomic study is needed to elucidate the mechanisms of these (BRRh-4 and BTL-M2) elite strains of probiotic bacteria on growth promotion, yield increase and microbial diversity in rice cultivated in the field conditions. Although the microbiome of rice roots and rhizosphere has become documented and discussed in several reports (Kim and Lee, 2020; Santos-Medellín et al., 2021), our study demonstrated that the application of two rice probiotic bacteria significantly improve the growth and yield of rice and modulate the bacterial diversity and population in roots and rhizosphere soils of treated rice plants. The performances of these bacteria on rice root and soil microbiome in different edaphic conditions should be evaluated for understanding the precise role in sustainable rice production with reduced chemical fertilizers.

## Conclusion

The microbiome inhabiting in rice roots and rhizosphere soils are taxonomically and phylogenetically diverse. Although they play a vital role in growth, health and productivity of rice, little is known about the interactions of rice plants and these microbial communities at a molecular level. In the current study, we demonstrated that two elite strains of plant probiotic bacteria, *P. fungorum* strain BRRh-4 and *Delftia* sp. strain BTL-M2 significantly improve seed germination and growth of rice plants both *in vitro* and *in planta*. In the pot experiments, both BRRh-4 and BTL-M2 with 50% reduced doses of recommended N, P, and K fertilizers produced almost equivalent yield of rice treated with 100% recommended doses of chemical fertilizers without application of bacteria. The amplicon sequencing analysis revealed that a combination of chemical fertilizers and *P. fungorum* strain BRRh-4 or *Delftia* sp. strain BTL-M2 significantly increased the diversity, structure and compositions of associated bacteriome in both in rice roots and rhizosphere soils. These probiotic bacteria may likely increase the availability and use efficiency of the N, P and K fertilizers by modulating rice root-associated microbiome. Our results indicate that application of BRRh-4 and BTL-M2 could reduce 50% N, P, and K fertilizers requirements for rice production without compromising the yield. Interestingly, we found the recruitment and/or abundance of specific bacterial genera in the rice treated with the specific probiotic bacteria which merits further investing to understand the molecular cross-talks in the rhizobiocomplex in rice. The novel findings of our study indicate that elite species of plant probiotic bacteria have tremendous potential for use as biofertilizers as an alternative to the costly and hazardous synthetic fertilizers, which are commonly used in conventional rice production. However, a large-scale field experiment and further metagenomics analyses are required for a better understanding of the effects of modulated microbiome on growth and yield of rice by the application of probiotic bacteria alone or in combination under reduced level of chemical fertilizers. The role of applied probiotic bacteria on health of rice cultivated soils also needs to be investigated.

## Data availability statement

The original contributions presented in the study are included in the article/Supplementary material, further inquiries can be directed to the corresponding author.

## Author contributions

TI conceived, designed and supervised the study. F, DRG and NUM carried out field and laboratory experiment, interpreted the results and drafted the manuscript. MNH and TIS executed the bioinformatics analysis, interpreted the results and drafted the manuscript. TI and AGS contributed intellectually to the interpretation and presentation of the results. All authors contributed to the article and approved the submitted version.

## Funding

This study was supported by the grant from Bangladesh Academy of Science (BAS)-USDA-PAL project CR-11 to TI.

## Conflict of interest

The authors declare that the research was conducted in the absence of any commercial or financial relationships that could be construed as a potential conflict of interest.

## Publisher’s note

All claims expressed in this article are solely those of the authors and do not necessarily represent those of their affiliated organizations, or those of the publisher, the editors and the reviewers. Any product that may be evaluated in this article, or claim that may be made by its manufacturer, is not guaranteed or endorsed by the publisher.
