# Supplementary material for: Improvement of growth, yield and associated bacteriome of rice by the application of probiotic Paraburkholderia and Delftia
Source: Front Microbiol. 2023 Jul 14;14:1212505. doi: 10.3389/fmicb.2023.1212505 (PMC10375411; doi:10.3389/fmicb.2023.1212505)
Supplement: Supplementary file 1 [file Data_Sheet_1.pdf]

Supplementary Figures

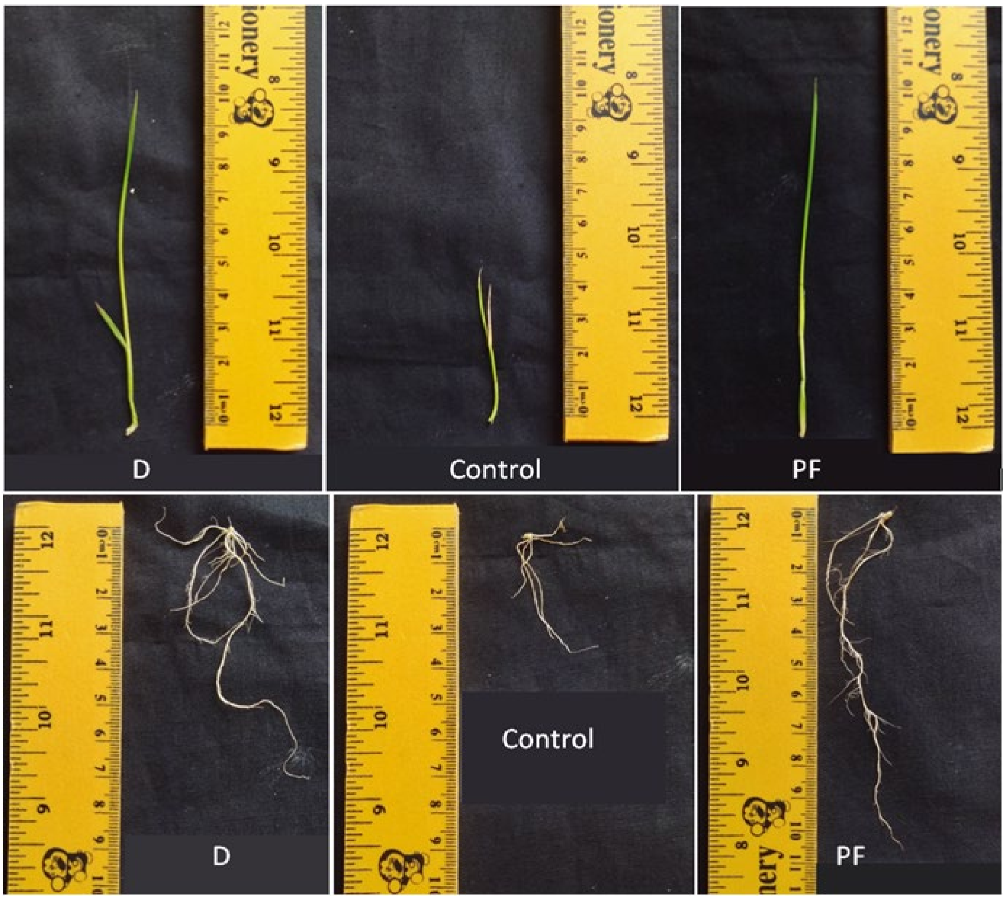

Figure S1: Representative shoot and root lengths of seedlings at 15 DAI in cm; randomly selected representative seedlings from both of the treatments and untreated controls showing the lengths in cm scale. D represents the seedlings treated with BTL-M2; Control is the untreated control seedlings and PF represents the seedlings treated with BRRh-4.

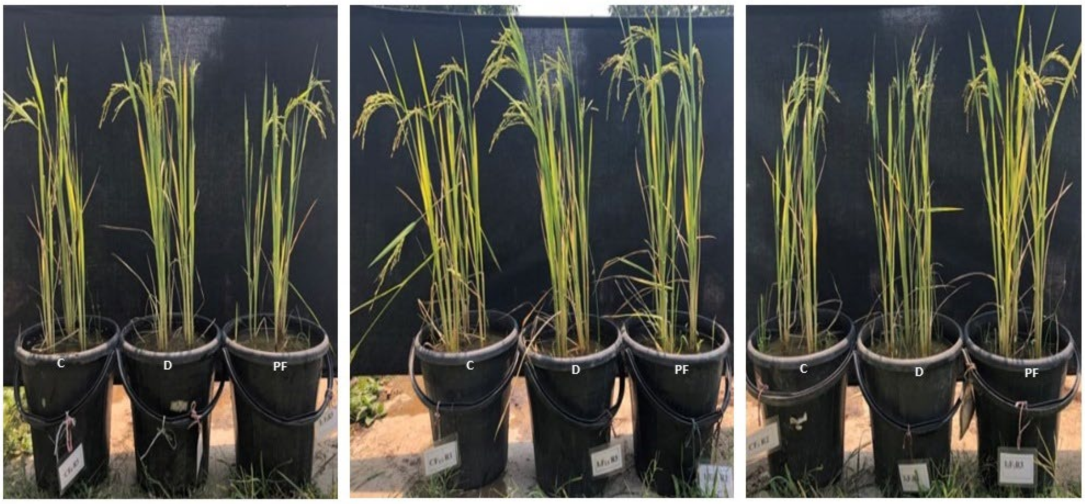

No fertilizer | Half doses of NPK fertilizers | Full doses of NPK fertilizers

Figure S2: Effect of BTL-M2 (D) and BRRh-4 (PF) on growth of rice under varying doses of recommended nitrogenous (N), phosphatic (P) and potassium (K) fertilizers. Here, C represents non-treated controls. Zero dose or no chemical fertilizers used. Half dose: 50% of recommended N, P, and K fertilizers used. Full dose: 100% of recommended N, P, and K fertilizers used.

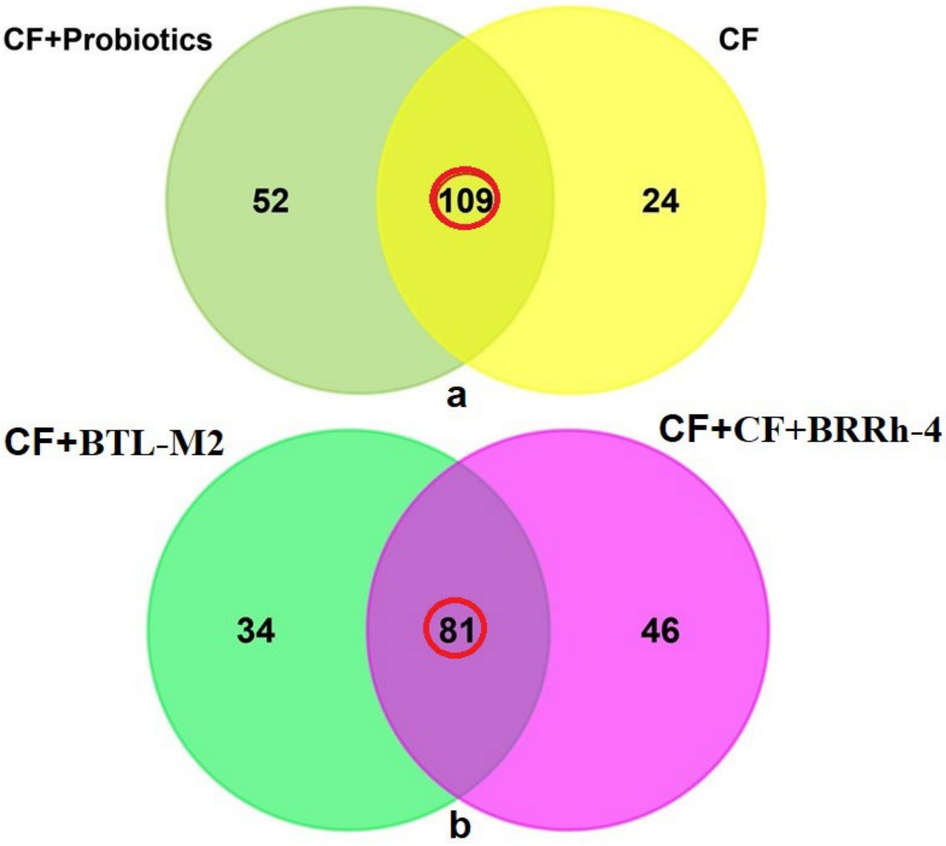

Figure S3: Microbiome diversity between chemical fertilizer (CF), and CF and probiotics bacteria treated metagenomes. (a) Venn diagram representing the unique and shared bacterial genera detected in these metagenomes. A total of 185 bacterial genera were detected in both metagenomes, and of them 109 genera were found to be common in the given conditions. (b) Microbiome diversity between CF + BTL-M2 and CF + BRRh-4 treated metagenomes. Venn diagram representing the unique and shared bacterial genera detected in CF + BTL-M2 and CF + BRRh-4 treated samples. A total of 161 bacterial genera were detected in both metagenomes where 81 genera were found to be shared between the two conditions.

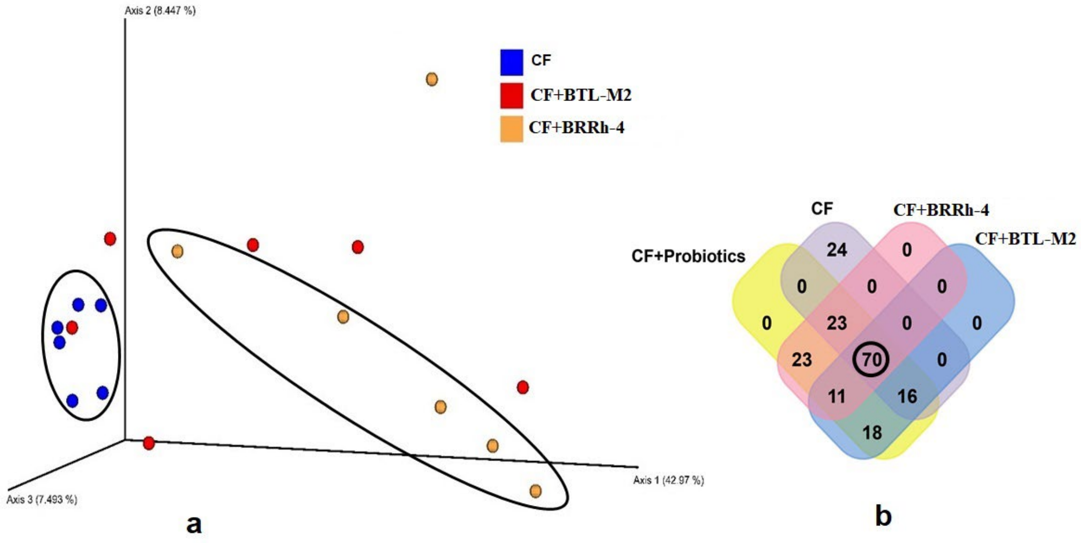

Figure S4: Taxonomic composition of microbiome in chemical fertilizer (CF), and CF + probiotics treatment (at genus level). (a) The PCA plot (beta diversity) based on Bray-Curtis metrics showed that most of the CF samples (blue colored) clustered closely (encircled in black) while the samples of CF + probiotics treated samples were distributed diversely (red and yellow colored) showing significant (Kruskal-Wallis test, $p = 0.002$) microbial disparity between the sample groups. However, among the probiotics treated samples more diversity differences were found in CF + BTL-M2 treated (red colored) sample group compared to CF + BRRh-4 treated (yellow colored) sample group (encircled in black). (b) Venn diagram comparison of bacterial genera in CF, CF + BTL-M2 and CF + BRRh-4 treated samples. Microbiome sharing between the conditions are indicated by black circles. More information on taxonomic composition and diversity can be found in Data S1.

Supplementary Tables

Table S1: Taxonomic distribution of microbiome at phylum level in root and rhizosphere soil metagenomes.

Phyla	Root	Rhizosphere soils
Bacteroidetes	42.910	67.254
Firmicutes	29.031	17.320
Proteobacteria	13.513	14.188
Planctomycetes	5.779	0.002
Thermi	4.151	0.004
Actinobacteria	2.015	1.125
Verrucomicrobia	1.076	0.093
Chloroflexi	0.875	0.00
Spirochaetes	0.184	0.00
Euryarchaeota	0.236	0.014
Nitrospirae	0.092	0.00
Cyanobacteria	0.140	0.00

Table S2: Taxonomic distribution of microbiome in root and rhizosphere soil metagenomes at order level.

Orders	Root	Rhizosphere soils
Bacteroidales	39.604	67.254
Bacillales	19.187	1.539
Clostridiales	9.437	15.473
Planctomycetales	5.213	0.002
Deinococcales	4.151	0.004
Flavobacteriales	3.157	0.000
Sphingomonadales	2.709	0.000
Vibrionales	2.563	6.272
Rhizobiales	2.513	0.077
Burkholderiales	1.798	1.369
Actinomycetales	1.682	1.125
Rhodospirillales	1.345	3.525
Aeromonadales	0.493	0.875
Enterobacteriales	0.762	0.558
Pseudomonadales	0.342	0.675
Pasteurellales	0.000	0.492
Chthoniobacterales	0.840	0.002
Erysipelotrichales	0.392	0.179
Alteromonadales	0.055	0.287
Gemmatales	0.485	0.000
Anaerolineales	0.438	0.000
Caldilineales	0.363	0.000
Bifidobacteriales	0.332	0.000
Xanthomonadales	0.239	0.031
Other orders	1.902	0.261

Table S3: Taxonomic distribution of top 30 bacterial genera in root and rhizosphere soil metagenomes.

Genera	Root (R)	Rhizosphere soil (S)
Prevotella	24.961	52.022
Unclassified	25.122	3.016
Bacteroides	2.607	12.383
Faecalibacterium	3.901	9.494
Bacillus	11.030	1.205
Vibrio	1.775	5.945
Roseomonas	0.814	3.391
Planctomyces	4.050	0.013
Deinococcus	2.961	0.021
Ruminococcus	0.384	1.762
Chryseobacterium	2.301	0.000
Exiguobacterium	1.909	0.000
Dialister	0.000	1.161
Novosphingobium	1.563	0.000
Sutterella	0.219	0.981
Butyrivibrio	0.000	1.066
Brevibacterium	0.338	0.801
Oscillospira	0.373	0.723
Succinivibrio	0.184	0.836
Megasphaera	0.142	0.665
Ancylobacter	0.908	0.074
Candidatus	0.750	0.002
Staphylococcus	0.500	0.126
Haemophilus	0.000	0.475
Corynebacterium	0.570	0.033
Parabacteroides	0.370	0.172
Massilia	0.596	0.000
Nocardioides	0.591	0.000
Enhydrobacter	0.208	0.282
Other genera	10.868	3.352
